# Association between COVID-19 by variant period and delivery-related outcomes in the United States

**DOI:** 10.1371/journal.pone.0355223

**Published:** 2026-08-12

**Authors:** Deborah Kilday, Michael Korvink, Drew Tatum, Zhun Cao, Ning Rosenthal, Raymond Perigard, Ashley Finke, Richelle Marshall, Margaret Snyder, Kelly Larson, Alain Moluh, Stephanie Martin, Samantha Sommerness, Craig Lipkin, Eyow Hodan, Dorothy A. Fink

**Affiliations:** 1 U.S. Department of Health & Human Services, Office on Women’s Health, Rockville, Maryland, United States of America; 2 Advisory Services, Premier, Inc., Charlotte, North Carolina, United States of America; Pescara General Hospital, ITALY

## Abstract

**Background:**

COVID-19 has been associated with adverse obstetric outcomes, but the magnitude of maternal and fetal risk may have varied across viral variant periods.

**Objective:**

To assess the association between COVID-19 infection and delivery-related in-hospital and 90-day mortality, severe maternal morbidity (SMM), fetal outcomes (fetal death and preterm birth), and a set of utilization measures (including cesarean delivery, ICU admission, costs, and hospital length of stay [LOS]) in the United States.

**Methods:**

A retrospective cohort study was conducted using delivery-related hospitalizations discharged between April 1, 2020, and March 31, 2023, from the Premier Healthcare Database. Regression modeling was conducted to evaluate variant-period-specific associations between COVID-19 exposure and key outcomes while controlling for a broad set of maternal risk factors.

**Results:**

Of the 2,561,615 evaluated delivery-related discharges, 53,226 (2.08%) were diagnosed with COVID-19. COVID-19 was associated with higher adjusted odds of in-hospital mortality across the Pre-Alpha, Alpha, Delta, and first Omicron phases (aOR [p-adj]: 13.11 [<.001], 13.03 [<.001], 29.47 [<.001], and 3.55 [.04]), respectively. Maternal COVID-19 infection was further associated with increased odds of fetal death at 20 or more weeks of gestation across the Pre-Alpha (aOR: 1.53 [1.28–1.83, < .001]) and Delta (3.37 [2.95–3.84, < .001]) phases.

The odds of experiencing any SMM, ICU admission, or preterm birth were significantly higher across all phases of the pandemic, whereas the odds of cesarean delivery were significantly higher during all but the Alpha and first Omicron phases. COVID-19 infection was associated with increased LOS and hospital costs throughout all variant phases.

**Conclusions:**

COVID-19 infection during delivery was associated with higher odds of maternal, fetal, and utilization outcomes at varying stages of the pandemic, with the most notable effect during the Delta period. These findings provide insight into the variant-dependent obstetric burden of COVID-19 and for maternal health surveillance and clinical preparedness during future infectious disease outbreaks.

## Introduction

As of May 5, 2023, when the World Health Organization (WHO) declared the end of the coronavirus disease 2019 (COVID-19) pandemic, more than 1.1 million deaths and nearly 7 million emergency department visits had been recorded in the United States (US) [1]. The COVID-19 pandemic caused significant disruptions to prenatal care, which were associated with increased risks of maternal and neonatal morbidity and mortality [2–10]. Previous studies reported cancellations of prenatal appointments, difficulties accessing prenatal classes, and changes to birth plans [11,12]. These disruptions were linked to elevated symptoms of depression and elevated pregnancy-related anxiety [11]. Population-level analyses revealed overall decreases in prenatal care visits and hospital birth rates, with increases in labor induction rates [13].

Regarding COVID-19 infection, a living systematic review by Allotey et al. reported higher odds of mortality, admission to an intensive care unit (ICU), invasive ventilation, cesarean delivery, and preterm birth were observed among delivering women with COVID-19 infection, along with higher odds of stillbirth and neonatal ICU admissions among neonates [14]. While systematic reviews and meta-analyses have provided valuable pooled estimates, these were primarily based on smaller observational studies with heterogeneous designs. There remains an opportunity to build upon prior research by leveraging a large, nationally diverse, multi-payer database with the comprehensive volume and granularity needed to more fully evaluate maternal and neonatal outcomes. Additionally, prior studies have typically evaluated COVID-19 exposure as a single dichotomous indicator, masking potential variant-period-specific risks. Finally, few have assessed incremental healthcare costs related to COVID-19 infection across distinct phases of the pandemic [10,15–18].

While the acute phase of the COVID-19 pandemic has passed, understanding the variant-specific obstetric burden remains critical for informing maternal health surveillance and clinical readiness for future infectious disease outbreaks. This study aimed to assess the association between COVID-19 infection and key delivery-related outcomes during periods corresponding to predominant COVID-19 variants among delivery-related hospitalizations occurring between April 1, 2020, and March 31, 2023, across 688 hospitals across the US. Maternal outcomes included severe maternal morbidity (SMM), a set of 20 unexpected outcomes of delivery indicators defined and maintained by the Centers for Disease Control (CDC) and Alliance for Innovation on Maternal Health (AIM), and mortality; utilization measures included cesarean delivery, ICU admission, hospital costs, and hospital length of stay (LOS); and fetal outcomes included fetal death and preterm birth [19].

## Methods

### Study design

A retrospective cohort study using data from the Premier Database (PHD) [20] was conducted to examine variant-period-specific associations between COVID-19 infection and in-hospital and 90-day episodic delivery-related outcomes in the US. The study was deemed exempt under 45 CFR 46.104(d)(4). It was conducted under WCG IRB Protocol No. 20261814 (Sponsor Protocol No. 75P00122R00055), for which a waiver of authorization for use of protected health information was approved on May 12, 2026. Data from the PHD are considered de-identified under HIPAA using the Expert Determination method. Informed consent was waived, as no direct contact with human subjects or identifiable information occurred. The study adhered to the principles of the Declaration of Helsinki and the Belmont Report.

### Data source

The PHD is a large, multi-payer, geographically diverse administrative database comprising not-for-profit, non-governmental, community, and teaching hospitals and health systems from rural and urban areas, representing approximately 20−25% of all inpatient admissions in the US [20]. The PHD contains International Classification of Diseases, Tenth Revision, Clinical Modification (ICD-10-CM) diagnoses, ICD-10 Procedure Coding System (ICD-10-PCS) procedures, and costs for billed hospital services for acute inpatient and outpatient hospital visits [20]. Patient- and visit-level elements are derived from uniform billing (UB-04) claims, in which missing or unavailable information is mapped to structured categories (e.g., unknown or information not available). For episodic outcomes, patient visits are linked using a hospital-specific unique patient identifier. Therefore, 90-day outcomes occurring outside of the delivery hospital were not observed.

### Study population

This study included delivery-related inpatient hospitalizations for women aged 10–64 years with discharge dates between April 1, 2020, and March 31, 2023, and a 90-day follow-up period through June 30, 2023. In alignment with AIM birth volume standards, deliveries were limited to those occurring in the inpatient setting, as identified by the presence of relevant Medicare Severity-Diagnosis-Related Groups (MS-DRG), ICD-10-CM diagnosis, or ICD-10-PCS procedure codes [21]. The full MS-DRG and ICD-10-based coding definitions used to identify deliveries in this study are provided in S1 Table.

Pregnancies indicating less than 20 weeks or unspecified gestation were excluded from the study population (n = 24,316), as pregnancy losses prior to 20 weeks of gestation are classified as miscarriages and therefore fall outside of the delivery-related hospitalization [22–24]. Hospitalizations with missing cost information (n = 2) were further excluded from the analysis. To account for anomalous or potentially erroneous data, hospitalization costs below the 1st percentile or above the 99th percentile were winsorized to their respective percentile thresholds. This means that extremely low and high values were replaced with the nearest values within the 1st and 99th percentiles, respectively, to limit the influence of outliers on the analysis. A total of 25,514 (0.996%) hospitalization costs were winsorized at the 1^st^ percentile, and 25,451 (0.994%) at the 99^th^ percentile. Additionally, 23,437 records with a LOS above the 99^th^ percentile were winsorized.

### Study variables

#### Exposure.

COVID-19 infection was identified through the presence of an ICD-10 diagnosis code of U07.1 recorded on the delivery hospitalization. The exposure of interest, variant-period-specific COVID-19 infection status, was defined as a set of binary indicators based on both the presence of a COVID-19 diagnosis during hospitalization and the predominant circulating COVID-19 variant at the time of hospitalization, as determined by CDC genomic surveillance data available beginning in January 2021. These indicators were constructed as a joint categorical variable combining patient-level COVID-19 diagnosis status with the predominant variant period corresponding to the patient’s discharge date. Predominant variant periods were defined as weeks during which the cumulative proportion of all Pango lineages belonging to a specific variant exceeded approximately 50% of the total weekly proportion. Variant period definitions were based on CDC data and aligned with publicly reported trends in variant predominance [25–29].

**Fig 1:**
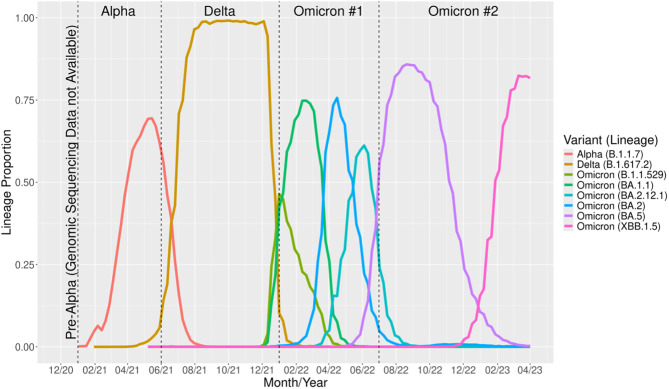
COVID-19 variant periods defined using genomic sequencing data.

The COVID-19 variant periods defined in this study included Pre-Alpha (April 1, 2020-December 31, 2020), Alpha (January 1, 2021-May 31, 2021), Delta (June 1, 2021-December 31, 2021), Omicron phase 1 (January 1, 2022-June 30, 2022), and Omicron phase 2 (July 1, 2022-March 31, 2023). Due to the distribution and duration of the Pango lineage, Omicron was divided into two variant phases. Additionally, the CDC’s collection period began in January 2021; therefore, all COVID-19 diagnoses occurring before this date were categorized as Pre-Alpha. The timeframes associated with each COVID-19 variant and a subset of the predominant lineages are illustrated in Fig 1.

To standardize exposure timing, only cases with a COVID-19 diagnosis documented during the delivery hospitalization were included in the exposed group. Infections occurring earlier in pregnancy but not documented at delivery were not captured in the analysis.

#### Outcomes.

A set of maternal, infant, and utilization outcomes was evaluated. This included maternal mortality, SMM, preterm birth, fetal death at ≥20 weeks of gestation, cesarean delivery, ICU admission, hospitalization cost, and LOS [30,31]. Maternal mortality was defined using the uniform billing discharge status code indicating an “expired” discharge.

SMM was assessed using a binary composite indicator reflecting the presence of any individual condition, with blood transfusion excluded per the most recent SMM specification [19]. The SMM list included acute myocardial infarction, acute renal failure, acute respiratory distress syndrome (ARDS), amniotic fluid embolism, aneurysm, cardiac arrest/ventricular fibrillation, conversion of cardiac rhythm, disseminated intravascular coagulation, eclampsia, heart failure/arrest during surgery or procedure, puerperal cerebrovascular disorders, pulmonary edema/acute heart failure, severe anesthesia complications, sepsis, shock, sickle cell disease with crisis, air and thrombotic embolism, hysterectomy, temporary tracheostomy, and ventilation [19]. The complete list of ICD-10 definitions for SMM is provided in S2 Table.

Ninety-day outcomes were evaluated for maternal outcomes (i.e., mortality and SMM), capturing events occurring during the index delivery admission and within the 90-day post-discharge period. Mortality and SMM were coded if they occurred during the index hospitalization or within 90 days of discharge, as is common practice for episodic outcome measures anchored to a hospital-based index visit [32,33].

Preterm birth, fetal death, and cesarean delivery were identified using ICD-10 diagnosis and MS-DRG codes (see S2 Table). ICU admissions were identified based on room and board charges associated with ICU-level care during the hospitalization. LOS was calculated as the number of days between the patient’s discharge and admission dates.

Costs were adjusted for inflation using the annual Consumer Price Index (CPI) from the US Bureau of Labor Statistics [34]. The final year of the study period, 2023, was used as the reference point, with costs from prior years updated to reflect inflation adjustments relative to the baseline.

#### Demographic, visit, and hospital characteristics.

Patient characteristics examined in this study included age, race, ethnicity, and primary insurance payer. Hospital characteristics included urban or rural status, American Hospital Association teaching status, the 9 US Census Bureau divisions, bed size, and annual birth volume. Payer categories were standardized into commercial insurance, Medicaid, charity or indigent care, Medicare, and other. Medicaid is government-funded insurance for individuals with low income, and Medicare is a federal health insurance program for individuals aged 65 and older or with specific disabilities. Bed size was categorized into three groups: 1–299, 300–499, and 500 or more beds. Hospital annual birth volume was grouped into four categories: 1–999, 1,000–1,999, 2,000–3,999, and 4,000 or more births.

Visit-level characteristics included patient admission type and admission source. Admission types for trauma, emergency, and urgent were collapsed into a single category due to low counts of trauma and emergency admissions.

#### Patient comorbidities.

A broad set of maternal risk factors was identified a priori through clinician review and further informed by established maternal risk indices, including the Maternal Comorbidity Index (MCI) [35] and Obstetric Comorbidity Scoring System (OCSS) [36]. Additional clinically-relevant risk factors beyond those captured within MCI and OCSS included assisted reproductive technology, cancer, chlamydia, connective tissue or autoimmune disease excluding lupus, cystic fibrosis, deep vein thrombosis, dialysis, gonococcal infection, granuloma inguinale, group B strep, hepatitis, herpes simplex, history tobacco use, other sexually transmitted infections (STI), polycystic ovary syndrome (PCOS), prior fetal death, supervision of high-risk pregnancy, syphilis, trichomoniasis, and uterine fibroids [37].

Conditions from the MCI and OCSS that had a known association with COVID-19 (e.g., preeclampsia [38]) were excluded from the comorbidity set to mitigate adjusting for factors that may be influenced by COVID-19. Anemia, group B strep, and pulmonary hypertension were only considered as comorbidities if present at the time of admission, to disambiguate preexisting conditions from those arising during the delivery hospitalization. Information on COVID-19 vaccination status was not available in the administrative data and was not included in the analyses. ICD-10 definitions for all maternal risk factors are provided in S3 Table.

A study flow diagram, illustrating the study inclusion and exclusion criteria and a graphical depiction of the study design, adapted from Schneeweiss et al., [39] are provided in S1 and S2 Figs, respectively.

### Statistical Analyses

Descriptive statistics were calculated by COVID-19 variant for patient and hospital characteristics, clinical covariates, and outcomes (Tables 1-3). Binary variables are reported as counts and percentages. Continuous variables are summarized using means, standard deviations, medians, and interquartile ranges (25th and 75th percentiles).

**Table 1:** Demographic, visit, and hospital characteristics stratified by COVID-19 variant period.

Characteristic	COVID-19 Not Documented N (%)	Pre-Alpha N (%)	Alpha N (%)	Delta N (%)	Omicron #1 N (%)	Omicron N (%)	Overall N (%)
Total N	2,508,389	11,751	6,990	9,397	17,848	7,240	2,561,615
Age Group, n (%)							
10-19	105,405 (4.2)	779 (6.6)	397 (5.7)	560 (6.0)	1,011 (5.7)	303 (4.2)	108,455 (4.2)
20-24	449,363 (18)	2,708 (23)	1,553 (22)	2,057 (22)	3,531 (20)	1,309 (18)	460,521 (18)
25-29	709,888 (28)	3,396 (29)	2,063 (30)	2,733 (29)	5,093 (29)	1,974 (27)	725,147 (28)
30-34	758,930 (30)	2,876 (24)	1,787 (26)	2,489 (26)	4,919 (28)	2,142 (30)	773,143 (30)
35-39	394,887 (16)	1,576 (13)	947 (14)	1,254 (13)	2,656 (15)	1,196 (17)	402,516 (16)
40-44	84,128 (3.4)	393 (3.3)	224 (3.2)	296 (3.1)	592 (3.3)	291 (4.0)	85,924 (3.4)
45-64	5,788 (0.2)	23 (0.2)	19 (0.3)	8 (<0.1)	46 (0.3)	25 (0.3)	5,909 (0.2)
Race, n (%)							
American Indian	22,164 (0.9)	125 (1.1)	57 (0.8)	96 (1.0)	193 (1.1)	71 (1.0)	22,706 (0.9)
Asian	120,207 (4.8)	347 (3.0)	288 (4.1)	254 (2.7)	755 (4.2)	454 (6.3)	122,305 (4.8)
Black	382,308 (15)	1,976 (17)	1,222 (17)	1,890 (20)	3,141 (18)	1,109 (15)	391,646 (15)
Other	225,507 (9.0)	2,167 (18)	784 (11)	911 (9.7)	2,028 (11)	899 (12)	232,296 (9.1)
Pacific Islander	23,077 (0.9)	170 (1.4)	92 (1.3)	82 (0.9)	175 (1.0)	51 (0.7)	23,647 (0.9)
Unable to Determine	138,775 (5.5)	1,044 (8.9)	477 (6.8)	474 (5.0)	1,107 (6.2)	570 (7.9)	142,447 (5.6)
White	1,596,351 (64)	5,922 (50)	4,070 (58)	5,690 (61)	10,449 (59)	4,086 (56)	1,626,568 (63)
Ethnicity, n (%)							
Hispanic or Latino	502,271 (20)	4,468 (38)	1,946 (28)	2,206 (23)	4,902 (27)	1,935 (27)	517,728 (20)
Not Hispanic or Latino	1,759,368 (70)	5,729 (49)	4,152 (59)	6,484 (69)	11,721 (66)	4,682 (65)	1,792,136 (70)
Unknown	246,750 (9.8)	1,554 (13)	892 (13)	707 (7.5)	1,225 (6.9)	623 (8.6)	251,751 (9.8)
Payer Type, n (%)							
Charity Indigent	2,395 (<0.1)	34 (0.3)	5 (<0.1)	22 (0.2)	26 (0.1)	12 (0.2)	2,494 (<0.1)
Commercial	1,325,338 (53)	3,978 (34)	2,885 (41)	3,942 (42)	7,763 (43)	3,394 (47)	1,347,300 (53)
Medicaid	1,040,798 (41)	7,048 (60)	3,759 (54)	4,956 (53)	9,172 (51)	3,438 (47)	1,069,171 (42)
Medicare	12,842 (0.5)	60 (0.5)	40 (0.6)	39 (0.4)	105 (0.6)	29 (0.4)	13,115 (0.5)
Other	127,016 (5.1)	631 (5.4)	301 (4.3)	438 (4.7)	782 (4.4)	367 (5.1)	129,535 (5.1)
Admission Type, n (%)							
Elective	1,327,401 (53)	5,622 (48)	3,253 (47)	4,141 (44)	8,728 (49)	3,726 (51)	1,352,871 (53)
Information Not Available	103,181 (4.1)	578 (4.9)	311 (4.4)	428 (4.6)	520 (2.9)	105 (1.5)	105,123 (4.1)
Trauma Center/Emergency/Urgent	1,077,807 (43)	5,551 (47)	3,426 (49)	4,828 (51)	8,600 (48)	3,409 (47)	1,103,621 (43)
Admission Source, n (%)							
Clinic	423,492 (17)	2,168 (18)	1,181 (17)	1,374 (15)	2,750 (15)	1,101 (15)	432,066 (17)
Information Not Available/Other	29,051 (1.2)	107 (0.9)	54 (0.8)	89 (0.9)	247 (1.4)	97 (1.3)	29,645 (1.2)
Non-Healthcare	2,025,764 (81)	9,295 (79)	5,588 (80)	7,653 (81)	14,544 (81)	5,902 (82)	2,068,746 (81)
Transfer	30,082 (1.2)	181 (1.5)	167 (2.4)	281 (3.0)	307 (1.7)	140 (1.9)	31,158 (1.2)
Rural or Urban, n (%)							
Rural	250,863 (10)	876 (7.5)	599 (8.6)	1,053 (11)	1,741 (9.8)	621 (8.6)	255,753 (10)
Urban	2,257,526 (90)	10,875 (93)	6,391 (91)	8,344 (89)	16,107 (90)	6,619 (91)	2,305,862 (90)
US Census Division, n (%)							
East North Central	356,760 (14)	1,312 (11)	887 (13)	1,381 (15)	2,360 (13)	1,090 (15)	363,790 (14)
East South Central	190,322 (7.6)	634 (5.4)	424 (6.1)	723 (7.7)	1,149 (6.4)	290 (4.0)	193,542 (7.6)
Mid Atlantic	288,990 (12)	2,282 (19)	1,150 (16)	999 (11)	2,339 (13)	1,703 (24)	297,463 (12)
Mountain	223,161 (8.9)	656 (5.6)	487 (7.0)	654 (7.0)	1,188 (6.7)	330 (4.6)	226,476 (8.8)
New England	92,777 (3.7)	256 (2.2)	215 (3.1)	232 (2.5)	578 (3.2)	391 (5.4)	94,449 (3.7)
Pacific	214,671 (8.6)	950 (8.1)	733 (10)	894 (9.5)	1,998 (11)	1,040 (14)	220,286 (8.6)
South Atlantic	687,911 (27)	2,933 (25)	1,769 (25)	2,652 (28)	5,107 (29)	1,541 (21)	701,913 (27)
West North Central	169,226 (6.7)	892 (7.6)	468 (6.7)	661 (7.0)	1,226 (6.9)	506 (7.0)	172,979 (6.8)
West South Central	284,571 (11)	1,836 (16)	857 (12)	1,201 (13)	1,903 (11)	349 (4.8)	290,717 (11)
Teaching Status, n (%)							
Non-Teaching Hospital	1,821,662 (73)	7,844 (67)	4,912 (70)	6,828 (73)	12,585 (71)	4,831 (67)	1,858,662 (73)
Teaching Hospital	686,727 (27)	3,907 (33)	2,078 (30)	2,569 (27)	5,263 (29)	2,409 (33)	702,953 (27)
Bed Size, n (%)							
1-299	930,785 (37)	3,460 (29)	2,227 (32)	3,263 (35)	6,467 (36)	2,377 (33)	948,579 (37)
300-499	729,694 (29)	3,740 (32)	2,207 (32)	2,833 (30)	5,205 (29)	2,313 (32)	745,992 (29)
500+	847,910 (34)	4,551 (39)	2,556 (37)	3,301 (35)	6,176 (35)	2,550 (35)	867,044 (34)
Birth Volume, n (%)							
1-999	369,647 (15)	1,467 (12)	1,074 (15)	1,578 (17)	2,701 (15)	1,039 (14)	377,506 (15)
1,000-1,999	581,338 (23)	2,324 (20)	1,479 (21)	2,096 (22)	3,962 (22)	1,602 (22)	592,801 (23)
2,000-3,999	920,359 (37)	4,675 (40)	2,708 (39)	3,497 (37)	6,939 (39)	2,602 (36)	940,780 (37)
4000+	637,045 (25)	3,285 (28)	1,729 (25)	2,226 (24)	4,246 (24)	1,997 (28)	650,528 (25)

**Table 3:** Maternal in-hospital outcomes stratified by COVID-19 variant period.

Outcome	COVID-19 Not Documented N (Rate/10,000)	Pre-Alpha N (Rate/10,000)	Alpha N (Rate/10,000)	Delta N (Rate/10,000)	Omicron #1 N (Rate/10,000)	Omicron #2 N (Rate/10,000)	Overall N (Rate/10,000)
Total N	2,508,389	11,751	6,990	9,397	17,848	7,240	2,561,615
Mortality	143 (0.57)	16 (14)	12 (17)	40 (43)	6 (3.4)	1 (1.4)	218 (0.85)
90-Day Mortality	211 (0.84)	16 (14)	12 (17)	44 (47)	6 (3.4)	1 (1.4)	290 (1.1)
SMM	20,264 (81)	530 (451)	387 (554)	993 (1,057)	354 (198)	135 (186)	22,663 (88)
90-Day SMM	28,953 (115)	578 (492)	423 (605)	1,069 (1,138)	443 (248)	159 (220)	31,625 (123)
Fetal Death	16,270 (65)	125 (106)	65 (93)	237 (252)	136 (76)	48 (66)	16,881 (66)
ICU Use	13,428 (54)	375 (319)	199 (285)	496 (528)	187 (105)	75 (104)	14,760 (58)
Cesarean Delivery	816,828 (3,256)	3,930 (3,344)	2,312 (3,308)	3,264 (3,473)	5,871 (3,289)	2,512 (3,470)	834,717 (3,259)
Preterm Delivery	98,111 (391)	606 (516)	393 (562)	618 (658)	890 (499)	402 (555)	101,020 (394)
Adjusted Cost							
Mean	9,116	10,374	10,345	11,033	9,848	10,769	9,142
SD	5,677	7,043	7,022	7,860	6,417	7,087	5,711
Median	7,648	8,411	8,348	8,518	8,041	8,758	7,661
(25%; 75%)	(5,533; 10,864)	(5,901; 12,220)	(5,955; 12,308)	(6,051; 12,956)	(5,788; 11,732)	(6,061; 12,982)	(5,540; 10,891)
Length of Stay							
Mean	2.43	2.55	2.63	2.77	2.51	2.61	2.43
SD	1.16	1.44	1.51	1.67	1.29	1.39	1.16
Median	2.00	2.00	2.00	2.00	2.00	2.00	2.00
(25%; 75%)	(2.00; 3.00)	(2.00; 3.00)	(2.00; 3.00)	(2.00; 3.00)	(2.00; 3.00)	(2.00; 3.00)	(2.00; 3.00)

Outcome-specific hierarchical generalized linear models (HGLMs) were fit using a set of dichotomous indicators for variant-period-specific COVID-19 exposure, with hospitalizations without a COVID-19 diagnosis serving as the reference group. The HGLM framework additionally controlled for patient and visit-level factors as fixed effects. Hospital characteristics (e.g., teaching status, region, and rurality) were modeled as random intercepts to account for heterogeneity associated with these facility-level factors. During early model development, models with hospital-specific random intercepts were also evaluated using a subset of facilities and produced nearly identical estimates for the primary exposure variables. Given the substantially increased model complexity and computational burden associated with including approximately 700 random intercepts in the full cohort, the more parsimonious specification utilizing key hospital characteristics was retained.

The frequency of control variables was descriptively analyzed by variant-period and COVID-19 status. Low-frequency variables without representation across all variant periods were excluded, resulting in the removal of assisted reproductive technology, cystic fibrosis, deep vein thrombosis, dialysis, and granuloma inguinale from the analysis. Multicollinearity was also assessed with Variance Inflation Factors (VIFs) [40]. VIFs were approximated using a simplified linear model that treated all covariates, including hospital characteristics, as fixed effects. All variables had VIFs below 2, indicating low multicollinearity; therefore, no variables were excluded on this basis.

For binary outcomes (i.e., mortality, SMM, fetal death, cesarean delivery, ICU admission, and preterm birth), logistic HGLMs were employed, while cost and LOS, as continuous outcomes, were modeled using a gamma distribution (log link) and Poisson distribution (log link), respectively [41]. Using a chi-square-based statistic, no substantive overdispersion was detected for LOS. As such, the Poisson rather than a negative binomial distribution was preferred. The coefficient for each variant-specific COVID-19 indicator was exponentiated to produce an adjusted odds ratio for binary outcomes, controlling for patient and hospital factors. Likewise, the COVID-19 coefficients for the cost- and LOS-specific HGLMs were exponentiated to evaluate the multiplicative association between COVID-19 infection type and the respective outcomes. A full description of the statistical modeling approach is available in the Supplementary Appendix in S2 File.

P-values were adjusted using the Holm-Bonferroni method to account for the family-wise error rate across all evaluated outcomes [42]. Significance for all analyses was determined at the.05 level.

### Sensitivity analysis

While the multilevel regression models adjusted for patient and hospital-level factors, they did not match non-COVID patients to variant periods, which may influence interpretation. To address this limitation, a sensitivity analysis was conducted using generalized additive models with the same covariate specification as the primary models, adding calendar time (measured in sequential calendar months) as a thin plate spline to flexibly account for temporal trends and evolving clinical practices. Adjusted odds ratios and exponentiated coefficients for variant-period exposure variables by outcome-specific GAM model are provided in S4 Table. Further, to assess the potential impact of variant misclassification during transition periods, monthly SMM rates among patients with COVID-19 were descriptively evaluated across these periods (S3 Fig).

All analyses were conducted using the R statistical software, Version 4.1.3 (R Foundation for Statistical Computing) [43]. The primary HGLM models and GAM models for sensitivity testing were implemented using the mgcv library [44].

## Results

Among the 2,561,615 delivery-related hospital discharges, 53,226 (2.08%) had a recorded COVID-19 diagnosis, with the percentage of patients having COVID-19 fluctuating by variant period (Pre-Alpha: 1.83%, Alpha: 1.75%, Delta: 2.09%, Omicron phase 1: 3.56%, Omicron phase 2: 1.27%). Among the 53,226 COVID-19 cases in the study period, the majority of patients were aged 25–34 years, with individuals aged 25–29 and 30–34 each accounting for 28–30% of the visits. Age distributions among patients with COVID-19 infection were relatively stable across variant periods and aligned with the overall population distributions, comprising both COVID-19 and non-COVID-19 cases. Notably, individuals aged 10–19 and 20–24 had a higher proportion of total COVID-19 cases during the Pre-Alpha (6.6% and 23%, respectively) than in the overall distribution (4.2% and 18%, respectively). Monthly SMM rates among patients with COVID-19 varied considerably across the study period (S3 Fig), increasing during the Alpha period (maximum of 837 per 10,000 in May 2021) and peaking during the Delta period (maximum of 1,360 per 10,000 deliveries in September 2021), before declining substantially during the Omicron phases (maximum of 264 per 10,000 deliveries in March 2023). SMM rates exhibited similar temporal patterns immediately before and after variant transition periods; the exception was the Delta-to-Omicron transition, where rates declined progressively over several months rather than transitioning abruptly at the boundary.

Maternal comorbidities differed between patients with and without COVID-19 during the study period. Acquired heart disease, anemia, chronic renal disease, obesity, and pre-existing bleeding disorder were consistently more common among patients with COVID-19 than those without. Rates of acquired heart disease, anemia, chronic renal disease, and pre-existing bleeding disorder were the highest during the Delta period (73, 137, 7.2, and 64 per 1,000 deliveries, respectively), while obesity peaked during the Alpha period (181 per 1,000 deliveries).

Racial and ethnic distributions among COVID-19 cases also varied modestly by variant period. The proportion of Black patients with COVID-19 was higher during the Delta period (20%) compared to the proportion of Black patients overall (15%). Patients with COVID-19 identifying as Hispanic or Latino were more frequently represented in the Pre-Alpha period (38%) compared to the overall proportion of Hispanic or Latino patients (20%).

Insurance coverage distributions among COVID-19 cases differed by variant period. Patients with Medicaid accounted for 60% of visits during Pre-Alpha, higher than the 42% of Medicaid cases overall.

Visit types remained relatively stable across periods, with elective admissions comprising around half of encounters (53% overall). However, trauma center/emergency/urgent admissions were slightly more common during the Delta period (51%) than overall (43%). Most visits originated from non-healthcare settings (81% overall), with minimal fluctuation across periods.

The rural-urban distribution was consistent across periods, with the majority of visits occurring in urban areas (90%). Regional representation varied slightly; for instance, the Mid-Atlantic region had a higher share of visits during Pre-Alpha and Omicron #2 (19% and 24%, respectively) compared to the overall average (12%).

### Association between COVID-19 and maternal outcomes

The associations between COVID-19 and key perinatal outcomes varied considerably at different points in the pandemic. After adjusting for patient and hospital characteristics, COVID-19 infection exhibited a positive statistically significant association with maternal in-hospital mortality across the Pre-Alpha, Alpha, Delta, and Omicron phase 1 (aOR [95% CI, p-value]: 13.11 [7.40–23.23, < .001], 13.03 [6.76–25.11, < .001], 29.47 [19.57–44.39, < .001], and 3.55 [1.49–8.45,.04]) respectively. Increased odds of 90-day mortality were significant during the Pre-Alpha, Alpha, and Delta phases (aOR: 10.09 [5.85–17.40, < .001], 10.11 [5.39–18.94, < .001], 25.11 [17.34–36.34, < .001]), respectively. There was insufficient evidence to establish an association between COVID-19 infection and 90-day mortality across the two Omicron phases, and in-hospital mortality was not statistically significant during the second Omicron phase.

The relationship between COVID-19 infection and in-hospital and 90-day SMM was significant across all variant periods with the Pre-Alpha, (5.55 [5.05–6.10, < .001] vs. 4.23 [3.87–4.62, < .001]), Alpha, (6.25 [5.57–7.00, < .001] vs. 4.81 [4.32–5.35, < .001]), Delta, (13.00 [12.06–14.01, < .001] vs. 9.64 [8.98–10.34, < .001]), Omicron phase 1, (2.17 [1.93–2.43, < .001] vs. 1.94 [1.75–2.14, < .001]), and Omicron phase 2, (1.86 [1.55–2.24, < .001] vs. 1.59 [1.35–1.88, < .001]).

COVID-19 was associated with increased odds of fetal death across the Pre-Alpha (aOR: 1.53 [1.28–1.83, < .001]) and Delta (3.37 [2.95–3.84, < .001]) phases; however, there was insufficient evidence to demonstrate a statistically significant association during the Alpha and Omicron phases. Similarly, COVID-19 during the Pre-Alpha (aOR: 1.10 [1.05–1.15,.001]) and Delta (1.20 [1.14–1.26, < .001]) phases was associated with increased odds of cesarean delivery in comparison to patients without a COVID-19 diagnosis across the study period.

COVID-19 was associated with relatively consistent elevated odds of preterm delivery across all stages of the pandemic (Pre-Alpha, aOR: 1.22 [1.12–1.32, < .001], Alpha, 1.29 [1.16–1.43, < .001], Delta, 1.42 [1.31–1.54, < .001], Omicron phase 1, 1.17 [1.09–1.25, < .001], and Omicron phase 2, 1.36 [1.23–1.51, < .001]). Patients positive for COVID-19 had the highest odds of being admitted to the ICU in the Pre-Alpha (5.88 [5.26–6.58, < .001]), Alpha, (4.28 [3.65–5.00, < .001]), and Delta (8.23 [7.42–9.13, < .001]) periods, with higher but reduced odds, during Omicron phase (1, 1.74 [1.49–2.03, < .001]), and Omicron phase 2 (1.88 [1.48–2.40, < .001]).

After adjusting for patient and hospital characteristics, COVID-19 was associated with a stable increase in cost throughout the pandemic. The exponentiated coefficients for in-hospital costs were Pre-Alpha (1.11 [1.10–1.12, < .001]), Alpha (1.09 [1.07–1.10, < .001]), Delta (1.17 [1.16–1.19, < .001]), Omicron phase 1 (1.05 [1.04–1.06, < .001]), and Omicron phase 2 (1.08 [1.07–1.10, < .001]). Consistent with the adjusted mean cost values in Table 3, the model results indicate that costs during hospitalization increased considerably (i.e., 16%−19% increase) during the Delta phase of the pandemic.

Patients with a recorded COVID-19 diagnosis also had modestly longer lengths of stay during hospitalization across the Pre-Alpha (1.04 [1.02–1.05, < .001]), Alpha (1.06 [1.05–1.08, < .001), Delta (1.11 [1.10–1.13, < .001]), Omicron phase 1 (1.02 [1.01–1.03, < .001]), and Omicron phase 2 (1.05 [1.04–1.07, < .001]) stages of the pandemic. These results translate to a 2%−11% mean increase in LOS for COVID-19 patients during hospitalization throughout the pandemic. The complete set of exponentiated coefficients for in-hospital and 90-day outcomes is provided in Table 4. Summary statistics for all model parameters are provided in S5–S14 Tables in S1 File. Results from the GAM sensitivity analyses were consistent with the primary models, with small differences in point estimates observed across outcomes and variant periods. Mortality estimates during the Pre-Alpha, Alpha, and Delta periods were modestly lower under the GAM specification, with confidence intervals largely overlapping those of the primary estimates (S4 Table).

**Table 4:** Adjusted odds ratios and exponentiated coefficients for COVID-19 exposure in regressions of maternal, infant, and utilization outcomes.

	Pre-Alpha		Alpha		Delta		Omicron #1		Omicron #2	
Outcome	Estimate (95% CI)	Adj. p-value	Estimate (95% CI)	Adj. p-value	Estimate (95% CI)	Adj. p-value	Estimate (95% CI)	Adj. p-value	Estimate (95% CI)	Adj. p-value
Mortality^1^	13.11 (7.40-23.23)	<.001	13.03 (6.76-25.11)	<.001	29.47 (19.57-44.39)	<.001	3.55 (1.49-8.45)	0.037	1.03 (0.12-8.86)	1
90-Day Mortality^1^	10.09 (5.85-17.40)	<.001	10.11 (5.39-18.94)	<.001	25.11 (17.34-36.34)	<.001	2.43 (1.03-5.73)	0.211	0.89 (0.12-6.85)	1
SMM^1^	5.55 (5.05-6.10)	<.001	6.25 (5.57-7.00)	<.001	13.00 (12.06-14.01)	<.001	2.17 (1.93-2.43)	<.001	1.86 (1.55-2.24)	<.001
90-Day SMM^1^	4.23 (3.87-4.62)	<.001	4.81 (4.32-5.35)	<.001	9.64 (8.98-10.34)	<.001	1.94 (1.75-2.14)	<.001	1.59 (1.35-1.88)	<.001
ICU Usage^1^	5.88 (5.26-6.58)	<.001	4.28 (3.66-5.00)	<.001	8.23 (7.42-9.13)	<.001	1.74 (1.49-2.03)	<.001	1.88 (1.48-2.40)	<.001
Fetal Death^1^	1.53 (1.28-1.83)	<.001	1.30 (1.02-1.67)	0.207	3.37 (2.95-3.84)	<.001	1.1 (0.92-1.30)	1	0.97 (0.73-1.29)	1
Preterm Delivery^1^	1.22 (1.12-1.32)	<.001	1.29 (1.16-1.43)	<.001	1.42 (1.31-1.54)	<.001	1.17 (1.09-1.25)	<.001	1.36 (1.23-1.51)	<.001
Cesarean Delivery^1^	1.10 (1.05-1.15)	0.001	1.08 (1.01-1.14)	0.112	1.20 (1.14-1.26)	<.001	1.05 (1.01-1.09)	0.112	1.16 (1.10-1.23)	<.001
Cost^2^	1.11 (1.10-1.12)	<.001	1.09 (1.07-1.10)	<.001	1.17 (1.16-1.19)	<.001	1.05 (1.04-1.06)	<.001	1.08 (1.07-1.10)	<.001
Length of Stay^2^	1.04 (1.02-1.05)	<.001	1.06 (1.05-1.08)	<.001	1.11 (1.10-1.13)	<.001	1.02 (1.01-1.03)	<.001	1.05 (1.04-1.07)	<.001

^1^Odds Ratio.

^2^Exponentiated Estimate.

## Discussion

Evaluating data through March 31, 2023, from a large, geographically diverse US hospital administrative database, this study found that COVID-19 was associated with significantly increased odds of maternal mortality, SMM, and other adverse outcomes among patients with in-hospital deliveries across all pandemic phases. COVID-19 diagnoses during the Delta period, however, resulted in the highest proportion of adverse outcomes, hospitalization costs, and prolonged hospital stays. Notably, Black women comprised 20% of patients with COVID-19 during this period, representing a 33% increase relative to their 15% representation in the overall study cohort. Additionally, the prevalence of comorbidities such as acquired heart disease, anemia, chronic renal disease, and pre-existing bleeding disorder was highest among those with COVID-19 during the Delta variant period.

While the Omicron phase 1 period had the highest proportion of COVID-19 diagnoses within the study period, Delta was associated with higher odds of maternal in-hospital and 90-day mortality, SMM, and fetal death at 20 or more weeks of gestation. COVID-19 was associated with a stable, significant increase in SMM incidence across all pandemic stages.

While COVID-19 was associated with increased odds of cesarean deliveries and preterm birth, the impact remained relatively stable across all variant phases.

The findings from this study are consistent with prior studies. In a previous study using data from the PHD from April 1, 2020, to November 23, 2020, Jering et al. [10] found that the diagnosis of COVID-19 at the time of delivery was associated with substantially higher odds of mortality (aOR: 26.1, 95% CI: 11.3–60.4), ICU admission, and moderately increased odds of preterm birth, stillbirth, and preeclampsia/eclampsia/HELLP (hemolysis, elevated liver enzymes, and low platelets) syndrome among 406,446 women. The current study extended the data period to March 2023 and included 2,561,615 delivery-related hospitalizations, 53,226 (2.08%) COVID-19 positive patients, and further provides greater specificity regarding the severity associated with specific variant clusters.

The markedly elevated risk of fetal death is consistent with prior research observing elevated preterm delivery risk across variant periods, most notably during the early stages of the pandemic [45–47]. Likewise, previous studies have observed an elevated risk of preterm birth associated with COVID-19 infection [48,49]. This study corroborates these findings using a broad, nationally-diverse, multi-payer database.

### Limitations

The study had several limitations. As with all administrative databases, identifying clinical conditions, procedures, and medications relies on the accuracy and completeness of the hospital-reported diagnosis, procedure codes, and hospital charge descriptions. This database was limited to inpatient delivery and postpartum re-admissions to the delivery hospital.

Linkage of patient records was restricted to individual facilities and, therefore, repeated delivery hospitalizations for the same patient at different hospitals were not captured. Given the duration of pregnancy, repeat deliveries within the study timeframe, even if occurring across hospitals, are expected to be uncommon and, as such, any unmeasured within-patient clustering would have a negligible impact on results.

COVID-19 diagnosed independent of the delivery-related admission could not be observed. Such under-detection may contribute to understated variant-period-specific risk. Furthermore, defining variant classification based on US surveillance data rather than patient-level sequencing may have introduced exposure misclassification, particularly during periods of variant co-circulation. Although descriptive evaluation of monthly SMM rates across transition periods generally demonstrated consistent temporal patterns, this approach does not quantify the extent of potential misclassification or its impact on exposure associations. Notably, the shift observed during the Delta to Omicron transition developed gradually, consistent with a true decline in disease severity rather than a misclassification artifact. Precise quantification of potential misclassification was not possible without patient-level sequencing data, which remains an important direction for future research, especially in the obstetric population.

Although a sensitivity analysis incorporating a flexible spline for calendar time demonstrated results highly consistent with the primary models, mortality point estimates differed the most during the Pre-Alpha and Alpha periods, though within overlapping confidence intervals of the primary estimates. This approach may not fully account for time-varying confounders, which may have had more pronounced effects earlier in the pandemic, such as changes in clinical management and healthcare system pressures. Given the evolving understanding of COVID-19 and its relationship with maternal outcomes, the potential for overadjustment or inappropriate adjustment of covariates remains. Additionally, controlling for hospital characteristics to account for hospital-level clustering may not capture the nuanced between-hospital variation that a hospital-specific random-intercept model would. However, exploratory models incorporating hospital-specific random intercepts produced nearly identical estimates for the primary COVID-19 exposure variables, suggesting that this modeling decision had minimal impact on the estimated associations between COVID-19 infection and study outcomes.

Furthermore, mortality was observed only in the hospital setting during the delivery-related admission and during the 90-day follow-up period. Deaths occurring outside of the index hospitalization and postpartum period were not captured, introducing potential information bias due to unobserved mortality events. Additionally, a key limitation of this study is the lack of patient-level COVID-19 vaccination status in the administrative data. Because vaccination may alter the risk of severe outcomes among pregnant patients with COVID-19, this limitation could introduce important unmeasured confounding [50].Vaccination may reduce the risk of severe outcomes, and its uptake likely varies across patient groups and time periods, potentially biasing comparisons between variant phases.

This study is limited to inpatient deliveries. Prior research has noted an increase in out-of-hospital births, including home births and births at birth centers, during the peak of the pandemic; however, as reported by the CDC, as of 2023, 97.68% of deliveries occurred in the hospital setting [51,52]. This may introduce selection bias, as patients who opted for home births may differ in characteristics and risk profiles from those who delivered in hospitals, potentially affecting the generalizability of the findings to the broader population of pregnant individuals during this period.

Finally, given the reliance on ICD-10 coding to determine patient COVID-19 status, it was not possible to distinguish between patients who tested negative for COVID-19 and those who were untested.

## Conclusion

This study examines the relationship between COVID-19 infection and maternal and infant outcomes, as well as healthcare resource utilization, across distinct phases of the pandemic defined by the predominant variants. While variant periods were assigned based on timing rather than patient-level sequencing data, the findings show consistently increased odds of adverse maternal outcomes, including mortality, severe maternal morbidity, fetal death ≥20 weeks, cesarean delivery, preterm birth, ICU admission, and prolonged hospitalization, across all phases, with the Delta period associated with the highest odds.

Moreover, the study highlights the increased economic burden associated with COVID-19 infection during pregnancy and childbirth, with hospitalization costs increasing by 17% and the length of stay extended by 11% during the Delta phase.

Overall, this study demonstrates that obstetric risk associated with COVID-19 was not static but evolved across variant periods, highlighting the need for ongoing maternal health surveillance and adaptive healthcare policies as infectious disease patterns change. These findings provide a framework for assessing and responding to variant-driven shifts in pregnancy risk during future infectious disease outbreaks. Further research is warranted to examine the long-term implications of COVID-19 infection during pregnancy and to inform evidence-based interventions to improve maternal and infant outcomes.

**Table 2:** Maternal comorbid conditions stratified by COVID-19 variant period.

Comorbidity	COVID-19 Not Documented N (Rate/1,000)	Pre-Alpha N (Rate/1,000)	Alpha N (Rate/1,000)	Delta N (Rate/1,000)	Omicron #1 N (Rate/1,000)	Omicron #2 N (Rate/1,000)	Overall N (Rate/1,000)
Total N	2,508,389	11,751	6,990	9,397	17,848	7,240	2,561,615
Acquired Heart Disease	119,861 (48)	632 (54)	425 (61)	682 (73)	1,080 (61)	472 (65)	123,152 (48)
Alcohol Use	1,942 (0.77)	9.0 (0.77)	3.0 (0.43)	8.0 (0.85)	12 (0.67)	11 (1.5)	1,985 (0.77)
Anemia	256,043 (102)	1,462 (124)	945 (135)	1,288 (137)	2,252 (126)	761 (105)	262,751 (103)
Asthma	163,552 (65)	669 (57)	472 (68)	784 (83)	1,289 (72)	585 (81)	167,351 (65)
Bariatric Surgery	15,282 (6.1)	61 (5.2)	56 (8.0)	56 (6.0)	101 (5.7)	65 (9.0)	15,621 (6.1)
Cancer	12,613 (5.0)	42 (3.6)	44 (6.3)	38 (4.0)	83 (4.7)	42 (5.8)	12,862 (5.0)
Cardiac Valvular Disease	3,516 (1.4)	11 (0.94)	11 (1.6)	24 (2.6)	36 (2.0)	15 (2.1)	3,613 (1.4)
Chlamydia	5,897 (2.4)	44 (3.7)	21 (3.0)	37 (3.9)	56 (3.1)	19 (2.6)	6,074 (2.4)
Chronic Ischemic Heart Disease	1,075 (0.43)	1.0 (0.09)	3.0 (0.43)	3.0 (0.32)	10 (0.56)	6.0 (0.83)	1,098 (0.43)
Chronic Renal Disease	6,719 (2.7)	43 (3.7)	40 (5.7)	68 (7.2)	69 (3.9)	37 (5.1)	6,976 (2.7)
Congenital Heart Disease	2,169 (0.86)	8.0 (0.68)	10 (1.4)	6.0 (0.64)	17 (0.95)	7.0 (0.97)	2,217 (0.87)
Connective Tissue or Autoimmune Disease Excluding Lupus	66,884 (27)	222 (19)	189 (27)	253 (27)	440 (25)	196 (27)	68,184 (27)
Current Tobacco Use	113,285 (45)	279 (24)	246 (35)	548 (58)	837 (47)	277 (38)	115,472 (45)
Gastrointestinal Disease Crohn’s Disease	116,748 (47)	438 (37)	357 (51)	510 (54)	811 (45)	364 (50)	119,228 (47)
Gonococcal	1,308 (0.52)	6.0 (0.51)	7.0 (1.0)	12 (1.3)	11 (0.62)	1.0 (0.14)	1,345 (0.53)
Group B Strep	449,991 (179)	2,118 (180)	1,275 (182)	1,585 (169)	3,213 (180)	1,267 (175)	459,449 (179)
Hepatitis	16,567 (6.6)	86 (7.3)	42 (6.0)	94 (10)	133 (7.5)	61 (8.4)	16,983 (6.6)
Herpes Simplex	82,191 (33)	318 (27)	224 (32)	309 (33)	614 (34)	294 (41)	83,950 (33)
History Tobacco Use	168,051 (67)	548 (47)	457 (65)	722 (77)	1,286 (72)	453 (63)	171,517 (67)
HIV/AIDS	2,217 (0.88)	22 (1.9)	9.0 (1.3)	8.0 (0.85)	23 (1.3)	12 (1.7)	2,291 (0.89)
Hyperthyroidism Graves Disease	10,542 (4.2)	32 (2.7)	32 (4.6)	36 (3.8)	87 (4.9)	32 (4.4)	10,761 (4.2)
Mental Health	283,996 (113)	842 (72)	718 (103)	1,135 (121)	1,964 (110)	893 (123)	289,548 (113)
Neuromuscular Disease	56,533 (23)	233 (20)	181 (26)	283 (30)	445 (25)	185 (26)	57,860 (23)
Obesity	399,501 (159)	2,092 (178)	1,268 (181)	1,680 (179)	3,169 (178)	1,299 (179)	409,009 (160)
Other STI	10,362 (4.1)	63 (5.4)	29 (4.1)	38 (4.0)	97 (5.4)	34 (4.7)	10,623 (4.1)
Polycystic Ovary Syndrome	26,159 (10)	87 (7.4)	60 (8.6)	56 (6.0)	151 (8.5)	89 (12)	26,602 (10)
Pre-existing Bleeding Disorder	72,115 (29)	501 (43)	333 (48)	601 (64)	629 (35)	272 (38)	74,451 (29)
Pre-existing Hypertension	94,822 (38)	356 (30)	250 (36)	344 (37)	668 (37)	293 (40)	96,733 (38)
Previous Cesarean	447,114 (178)	2,163 (184)	1,206 (173)	1,595 (170)	3,175 (178)	1,248 (172)	456,501 (178)
Prior Fetal Death	6,741 (2.7)	22 (1.9)	10 (1.4)	28 (3.0)	46 (2.6)	14 (1.9)	6,861 (2.7)
Pulmonary Hypertension	613 (0.24)	4.0 (0.34)	6.0 (0.86)	14 (1.5)	5.0 (0.28)	1.0 (0.14)	643 (0.25)
Seizure Disorders	15,388 (6.1)	72 (6.1)	48 (6.9)	99 (11)	112 (6.3)	44 (6.1)	15,763 (6.2)
Sickle Cell	19,411 (7.7)	106 (9.0)	63 (9.0)	77 (8.2)	161 (9.0)	65 (9.0)	19,883 (7.8)
Substance Use	72,347 (29)	249 (21)	218 (31)	421 (45)	618 (35)	221 (31)	74,074 (29)
Supervision of High-Risk Pregnancy	10,544 (4.2)	56 (4.8)	33 (4.7)	51 (5.4)	83 (4.7)	32 (4.4)	10,799 (4.2)
Syphilis	3,870 (1.5)	18 (1.5)	9.0 (1.3)	22 (2.3)	44 (2.5)	27 (3.7)	3,990 (1.6)
Systemic Lupus Erythematosus	4,268 (1.7)	19 (1.6)	15 (2.1)	15 (1.6)	40 (2.2)	15 (2.1)	4,372 (1.7)
Trichomoniasis	4,090 (1.6)	17 (1.4)	14 (2.0)	32 (3.4)	49 (2.7)	16 (2.2)	4,218 (1.6)
Type I Pre-existing Diabetes	6,161 (2.5)	47 (4.0)	23 (3.3)	28 (3.0)	41 (2.3)	12 (1.7)	6,312 (2.5)
Type II Pre-existing Diabetes	15,713 (6.3)	143 (12)	84 (12)	74 (7.9)	112 (6.3)	65 (9.0)	16,191 (6.3)
Uterine Fibroids	49,720 (20)	229 (19)	124 (18)	177 (19)	357 (20)	195 (27)	50,802 (20)

## Supporting information

S1 Fig Flow diagram of study cohort selection for inpatient deliveries during the COVID-19 pandemic. (PNG)

S2 Fig Temporal structure of the study design including exposure, covariate assessment, and outcome evaluation windows. (PNG)

S3 Fig Monthly SMM rates among patients with COVID-19 across variant transition periods. (PNG)

S1 Table ICD-10 and MS-DRG cohort definitions for study inclusion. (DOCX)

S2 Table ICD-10 maternal outcome definitions. (DOCX)

S3 Table ICD-10 maternal risk factor definitions. (DOCX)

S4 Table Adjusted odds ratios and exponentiated coefficients from generalized additive models incorporating time-series splines to assess variant-period effects. (DOCX)

S1 File Supplemental table 5–14. Regression coefficient summary by outcome type.
(DOCX)

S2 File COVID_19 statistical supplement. (DOCX)
