# Supplementary material for: Association between COVID-19 by variant period and delivery-related outcomes in the United States
Source: PLoS One. 2026 Aug 12;21(8):e0355223. doi: 10.1371/journal.pone.0355223 (PMC13466025; doi:10.1371/journal.pone.0355223)
Supplement: Supplementary file 1 [file pone.0355223.s004.docx]

**Supplemental Table 1. ICD-10 and MS-DRG Cohort Definitions for Study Inclusion**

| **ICD-10-CM** | **Descriptions** |
| --- | --- |
| **Delivery Diagnosis** |  |
| Z37.0 | Single live birth |
| Z37.1 | Single stillbirth |
| Z37.2 | Twins, both liveborn |
| Z37.3 | Twins, one liveborn and one stillborn |
| Z37.4 | Twins, both stillborn |
| Z37.50 | Multiple births, unspecified, all liveborn |
| Z37.51 | Triplets, all liveborn |
| Z37.52 | Quadruplets, all liveborn |
| Z37.53 | Quintuplets, all liveborn |
| Z37.54 | Sextuplets, all liveborn |
| Z37.59 | Other multiple births, all liveborn |
| Z37.60 | Multiple births, unspecified, some liveborn |
| Z37.61 | Triplets, some liveborn |
| Z37.62 | Quadruplets, some liveborn |
| Z37.63 | Quintuplets, some liveborn |
| Z37.64 | Sextuplets, some liveborn |
| Z37.69 | Other multiple births, some liveborn |
| Z37.7 | Other multiple births, all stillborn |
| Z37.9 | Outcome of delivery, unspecified |
| O75.82 | Onset (spontaneous) of labor after 37 completed weeks of gestation but before 39 completed weeks gestation, with delivery by (planned) cesarean section |
| O80 | Encounter for full-term uncomplicated delivery |
| O82 | Encounter for cesarean delivery without indication |
| **Delivery Procedure** |  |
| 10D00Z0 | Extraction of Products of Conception, Classical, Open Approach |
| 10D00Z1 | Extraction of Products of Conception, Low Cervical, Open Approach |
| 10D00Z2 | Extraction of Products of Conception, Extraperitoneal, Open Approach |
| 10D07Z3 | Extraction of Products of Conception, Low Forceps, Via Natural or Artificial Opening |
| 10D07Z4 | Extraction of Products of Conception, Mid Forceps, Via Natural or Artificial Opening |
| 10D07Z5 | Extraction of Products of Conception, High Forceps, Via Natural or Artificial Opening |
| 10D07Z6 | Extraction of Products of Conception, Vacuum, Via Natural or Artificial Opening |
| 10D07Z7 | Extraction of Products of Conception, Internal Version, Via Natural or Artificial Opening |

| 10D07Z8 | Extraction of Products of Conception, Other, Via Natural or Artificial Opening |
| --- | --- |
| 10E0XZZ | Delivery of Products of Conception, External Approach |
|  |  |
| **Delivery DRG** |  |
| 765 | Cesarean section w cc/mcc |
| 766 | Cesarean section w/o cc/mcc |
| 767 | Vaginal delivery w sterilization &/or d&c |
| 768 | Vaginal delivery w o.r. proc except steril &/or d&c |
| 774 | Vaginal delivery w complicating diagnoses |
| 775 | Vaginal delivery w/o complicating diagnoses |
| 783 | Cesarean section w sterilization w mcc |
| 784 | Cesarean section w sterilization w cc |
| 785 | Cesarean section w sterilization w/o cc/mcc |
| 786 | Cesarean section w/o sterilization w mcc |
| 787 | Cesarean section w/o sterilization w cc |
| 788 | Cesarean section w/o sterilization w/o cc/mcc |
| 796 | Vaginal delivery w sterilization/d&c w mcc |
| 797 | Vaginal delivery w sterilization/d&c w cc |
| 798 | Vaginal delivery w sterilization/d&c wo cc/mcc |
| 805 | Vaginal delivery without sterilization/d&c with mcc |
| 806 | Vaginal delivery without sterilization/d&c with cc |
| 807 | Vaginal delivery without sterilization/d&c without cc/mcc |
