# Supplementary material for: Association between COVID-19 by variant period and delivery-related outcomes in the United States
Source: PLoS One. 2026 Aug 12;21(8):e0355223. doi: 10.1371/journal.pone.0355223 (PMC13466025; doi:10.1371/journal.pone.0355223)
Supplement: Supplementary file 2 [file pone.0355223.s005.docx]

**Supplemental Table 2. ICD-10 Maternal Outcome Definitions**

| **Fetal Death Diagnosis** | |  | |
| --- | --- | --- | --- |
| O36.4XX0 | | Maternal care for intrauterine death, not applicable or unspecified | |
| O36.4XX1 | | Maternal care for intrauterine death, fetus 1 | |
| O36.4XX2 | | Maternal care for intrauterine death, fetus 2 | |
| O36.4XX3 | | Maternal care for intrauterine death, fetus 3 | |
| O36.4XX4 | | Maternal care for intrauterine death, fetus 4 | |
| O36.4XX5 | | Maternal care for intrauterine death, fetus 5 | |
| O36.4XX9 | | Maternal care for intrauterine death, other fetus | |
| Z37.1 | | Single stillbirth | |
| Z37.3 | | Twins, one liveborn and one stillborn | |
| Z37.4 | | Twins, both stillborn | |
| Z37.7 | | Other multiple births, all stillborn | |
| Z37.60 | | Multiple births, unspecified, some liveborn | |
| Z37.61 | | Triplets, some liveborn | |
| Z37.62 | | Quadruplets, some liveborn | |
| Z37.63 | | Quintuplets, some liveborn | |
| Z37.64 | | Sextuplets, some liveborn | |
| Z37.69 | | Other multiple births, some liveborn | |
| **Cesarean Delivery MS-DRG** | |  | |
| 765 | | Cesarean section w cc/mcc | |
| 766 | | Cesarean section w/o cc/mcc | |
| 783 | | Cesarean section w sterilization w mcc | |
| 784 | | Cesarean section w sterilization w cc | |
| 785 | | Cesarean section w sterilization w/o cc/mcc | |
| 786 | | Cesarean section w/o sterilization w mcc | |
| 787 | | Cesarean section w/o sterilization w cc | |
| 788 | | Cesarean section w/o sterilization w/o cc/mcc | |
| **Preterm Birth Diagnosis** | |  | |
| O60.00 | | Preterm labor without delivery unspecified trimester | |
| O60.02 | | Preterm labor without delivery second trimester | |
| O60.03 | | Preterm labor without delivery third trimester | |
|  | |  | |
| O60.10X0 | | Preterm labor with preterm delivery, unspecified trimester not applicable or unspecified | |
| O60.10X1 | | Preterm labor with preterm delivery, unspecified trimester fetus 1 | |
| O60.10X2 | | Preterm labor with preterm delivery, unspecified trimester fetus 2 | |
| O60.10X3 | | Preterm labor with preterm delivery, unspecified trimester fetus 3 | |
| O60.10X4 | | Preterm labor with preterm delivery, unspecified trimester fetus 4 | |
| O60.10X5 | | Preterm labor with preterm delivery, unspecified trimester fetus 5 | |
| O60.10X9 | | Preterm labor with preterm delivery, unspecified trimester other fetus | |
| O60.12 | | Preterm labor second trimester with preterm delivery second trimester | |
| O60.12X0 | | Preterm labor second trimester with preterm delivery second trimester not applicable or unspecified | |
| O60.12X1 | | Preterm labor second trimester with preterm delivery second trimester fetus 1 | |
| O60.12X2 | | Preterm labor second trimester with preterm delivery second trimester fetus 2 | |
| O60.12X3 | | Preterm labor second trimester with preterm delivery second trimester fetus 3 | |
| O60.12X4 | | Preterm labor second trimester with preterm delivery second trimester fetus 4 | |
| O60.12X5 | | Preterm labor second trimester with preterm delivery second trimester fetus 5 | |
| O60.12X9 | | Preterm labor second trimester with preterm delivery second trimester other fetus | |
| O60.13 | | Preterm labor second trimester with preterm delivery third trimester | |
| O60.13X0 | | Preterm labor second trimester with preterm delivery third trimester not applicable or unspecified | |
| O60.13X1 | | Preterm labor second trimester with preterm delivery third trimester fetus 1 | |
| O60.13X2 | | Preterm labor second trimester with preterm delivery third trimester fetus 2 | |
| O60.13X3 | | Preterm labor second trimester with preterm delivery third trimester fetus 3 | |
| O60.13X4 | | Preterm labor second trimester with preterm delivery third trimester fetus 4 | |
| O60.13X5 | | Preterm labor second trimester with preterm delivery third trimester fetus 5 | |
| O60.13X9 | | Preterm labor second trimester with preterm delivery third trimester other fetus | |
| O60.14 | | Preterm labor third trimester with preterm delivery third trimester | |
| O60.14X0 | | Preterm labor third trimester with preterm delivery third trimester not applicable or unspecified | |
| O60.14X1 | | Preterm labor third trimester with preterm delivery third trimester fetus 1 | |
| O60.14X2 | | Preterm labor third trimester with preterm delivery third trimester fetus 2 | |
| O60.14X3 | | Preterm labor third trimester with preterm delivery third trimester fetus 3 | |
| O60.14X4 | | Preterm labor third trimester with preterm delivery third trimester fetus 4 | |
| O60.14X5 | | Preterm labor third trimester with preterm delivery third trimester fetus 5 | |
| O60.14X9 | | Preterm labor third trimester with preterm delivery third trimester other fetus | |
| O60.2 | | Term delivery with preterm labor | |
| O60.20 | | Term delivery with preterm labor, unspecified trimester | |
| O60.20X0 | | Term delivery with preterm labor, unspecified trimester not applicable or unspecified | |
| O60.20X1 | | Term delivery with preterm labor, unspecified trimester fetus 1 | |
| O60.20X2 | | Term delivery with preterm labor, unspecified trimester fetus 2 | |
| O60.20X3 | | Term delivery with preterm labor, unspecified trimester fetus 3 | |
| O60.20X4 | | Term delivery with preterm labor, unspecified trimester fetus 4 | |
| O60.20X5 | | Term delivery with preterm labor, unspecified trimester fetus 5 | |
| O60.20X9 | | Term delivery with preterm labor, unspecified trimester other fetus | |
| O60.22 | | Term delivery with preterm labor, second trimester | |
| O60.22X0 | | Term delivery with preterm labor, second trimester not applicable or unspecified | |
| O60.22X1 | | Term delivery with preterm labor, second trimester fetus 1 | |
| O60.22X2 | | Term delivery with preterm labor, second trimester fetus 2 | |
| O60.22X3 | | Term delivery with preterm labor, second trimester fetus 3 | |
| O60.22X4 | | Term delivery with preterm labor, second trimester fetus 4 | |
| O60.22X5 | | Term delivery with preterm labor, second trimester fetus 5 | |
| O60.22X9 | | Term delivery with preterm labor, second trimester other fetus | |
| O60.23 | | Term delivery with preterm labor, third trimester | |
| O60.23X0 | | Term delivery with preterm labor, third trimester not applicable or unspecified | |
| O60.23X1 | | Term delivery with preterm labor, third trimester fetus 1 | |
| O60.23X2 | | Term delivery with preterm labor, third trimester fetus 2 | |
| O60.23X3 | | Term delivery with preterm labor, third trimester fetus 3 | |
| O60.23X4 | | Term delivery with preterm labor, third trimester fetus 4 | |
| O60.23X5 | | Term delivery with preterm labor, third trimester fetus 5 | |
| **SMM** | |  | |
| Acute Myocardial Infarction  Aneurysm  Acute Renal Failure  Acute Respiratory Distress Syndrome  Amniotic Fluid Embolism  Cardiac Arrest / Ventricular Fibrillation  Conversion of Cardiac Rhythm  Disseminated Intravascular Coagulation  Eclampsia  Heart Failure / Arrest During Surgery or Procedure  Puerperal Cerebrovascular Disorders  Pulmonary Edema / Acute Heart Failure  Severe Anesthesia Complications  Sepsis  Shock  Sickle Cell Disease With Crisis  Air and Thrombotic Embolism  Hysterectomy  Temporary Tracheostomy  Ventilation | I21.xx, I22.x  I71.xx, I79.0  N17.x, O90.4  J80, J95.1, J95.2, J95.3, J95.82x, J96.0x, J96.2x, J96.9x, R06.03, R09.2  O88.112, O88.113, O88.119, O88.12, O88.13  I46.x, I49.0x  5A12012, 5A2204Z  D65, D68.8, D68.9, O45.002, O45.003, O45.009, O45.012, O45.013, O45.019, O45.022, O45.023, O45.029, O45.092, O45.093, O45.099, O46.002, O46.003, O46.009, O46.012, O46.013, O46.019, O46.022, O46.023, O46.029, O46.092, O46.093, O46.099, O67.0, O72.3  O15. X  I97.120, I97.121, I97.130, I97.131, I97.710, I97.711  A81.2, G45.x, G46.x, G93.49, H34.0x, I60.xx, I61.xx, I62.xx, I63.00, I63.01x, I63.1xx, I63.2xx, I63.3xx, I63.4xx, I63.5xx, I63.6, I63.8x, I63.9, I65.xx, I66.xx, I67.xx, I68.xx, O22.50, O22.52, O22.53, I97.810, I97.811, I97.820, I97.821, O87.3  I50.1, I50.20, I50.21, I50.23, I50.30, I50.31, I50.33, I50.40, I50.41, I50.43, I50.810, I50.811, I50.813, I50.814, I50.82, I50.83, I50.84, I50.89, I50.9, J81.0  O29.112–O29.119, O29.122–O29.129, O29.192–O29.199, O29.212–O29.219, O29.292–O29.299, O74.0, O74.1, O74.2, O74.3, O89.0x, O89.1, O89.2, T88.2XXA, T88.3XXA  A32.7, A40.x, A41.x, I76, O85, O86.04, R65.20, R65.21, T81.12XA, T81.44XA  O75.1, R57.x, T78.2XXA, T81.10XA, T81.11XA, T81.19XA, T88.6XXA  D57.00, D57.01, D57.02, D57.211, D57.212, D57.219, D57.411, D57.412, D57.419, D57.811, D57.812, D57.819  I26.x, O88.012–O88.03, O88.212–O88.23, O88.312–O88.33, O88.812–O88.83, T80.0XXA  0UT90ZL, 0UT90ZZ, 0UT97ZL, 0UT97ZZ  0B110F4, 0B113F4, 0B114F4  5A1935Z, 5A1945Z, 5A1955Z | |  |
