# Supplementary material for: Association between COVID-19 by variant period and delivery-related outcomes in the United States
Source: PLoS One. 2026 Aug 12;21(8):e0355223. doi: 10.1371/journal.pone.0355223 (PMC13466025; doi:10.1371/journal.pone.0355223)
Supplement: Supplementary file 3 [file pone.0355223.s006.docx]

Supplemental Table 3. ICD-10 Maternal Risk Factor Definitions

| **Condition** | **Codes** |
| --- | --- |
| **Acquired Heart Disease** | A32.82, A36.81, A38.1, A39.51-A39.52, A52.03, B26.82, B33.21-B33.22, B33.24, B58.81, D86.85, I00, I01.1-I01.2, I01.8-I01.9, I02, I09, I21.B, I24.8-I24.9, I27.1, I27.22, I27.89, I30, I31.0-I31.1, I31.8-I31.9, I32-I33, I35-I45, I47-I48, I49.1-I49.5, I49.8-I49.9, I50.22, I50.32, I50.42, I50.812, I51.4-I51.5, I51.7, I51.89, I51.9, I52, I5A, J10.82, J11.82, M05.3, O90.3, O99.4, P09.5, R00-R01, R03, Z82.49 |
| **Alcohol Use** | F10.10-F10.12, F10.14-F10.15, F10.18-F10.19, F10.2, F10.92, F10.94-F10.97, F10.980, F10.982, F10.988, F10.99 |
| **Anemia** | D46.0-D46.2, D46.4, D50-D53, D55.0-D55.1, D55.21, D55.29, D55.3, D55.8-D55.9, D56, D58, D59.0, D59.10-D59.13, D59.19, D59.2, D59.30-D59.32, D59.39, D59.4-D59.6, D59.8-D59.9, D60, D61.01, D61.09, D61.1-D61.3, D61.8-D61.9, D63-D64, O90.81, O99.01, O99.03 |
| **Asthma** | J45 |
| **Bariatric Surgery** | O99.84, Z98.84 |
| **Cancer** | C00-C26, C30-C34, C37-C41, C43.0, C43.10, C43.111-C43.112, C43.121-C43.122, C43.2-C43.9, C44-C49, C4A, C50-C58, C60-C79, C7A, C7B, C80-C86, C88, C90-C96, D00-D07, D09, D37-D45, D46.9, D46.A, D46.B, D46.C, D46.Z, D47.0-D47.1, D47.3-D47.4, D47.9, D47.Z9, D48-D49, D70.1, G13.0, G89.3, I31.31, J91.0, M36.0-M36.1, M84.5, M90.6, O9A.1, R18.0, R53.0, R97.21, Z08, Z85, Z92.21 |
| **Cardiac Valvular Disease** | I05-I08, I34.0-I34.2, I34.81, I34.89, I34.9 |
| **Chlamydia** | A55-A56, A74, J16.0 |
| **Chronic Ischemic Heart Disease** | I20.0-I20.2, I20.81, I20.89, I20.9, I25.1-I25.7, I25.81-I25.84, I25.89, I25.9 |
| **Chronic Renal Disease** | N03, N04.0-N04.1, N04.20-N04.22, N04.29, N04.3-N04.9, N04.A, N05, N18.1-N18.2, N18.30-N18.32, N18.4-N18.6, N18.9, N25, N26.9, O26.83 |
| **Congenital Heart Disease** | Q20, Q21.0, Q21.10-Q21.16, Q21.19-Q21.23, Q21.3-Q21.4, Q21.8-Q21.9, Q22-Q24 |
| **Connective Tissue or Autoimmune Disease Excluding Lupus** | B57.5, C49.0-C49.6, C49.8-C49.9, D21, D69.0, D69.3, D69.41, D75.81, D80, D81.0-D81.2, D81.30-D81.32, D81.39, D81.4-D81.9, D82-D84, D86.0-D86.3, D86.81-D86.84, D86.86-D86.87, D86.89, D86.9, D89, E06.3, E23.6, E31.0, E71.528, E78.5, G04.0, G04.81, G25.3, G35, G36.0-G36.1, G47.4, G51.0, G51.8, G54.5, G61.0, G61.81-G61.82, G70.0, G70.80-G70.81, G72.4, G90.9, G90.A, G93.32, H10.51, H16.05, H16.321, H30.10, H81.0, H83.8, H91.2, I24.1, I51.4, I73.0, J84.112, K29.4, K63.8, K65.4, K74.3, K83.01, K86.1, K90.0, L10, L12.0-L12.1, L13.0, L13.8-L13.9, L14, L30.8, L40, L43, L50.0, L57.4, L65.9, L73.2, L90-L92, L94-L95, L98-L99, M12.3, M30.0-M30.1, M30.3, M31.0, M31.3-M31.4, M31.6-M31.9, M33, M34.1, M35.0, M35.2-M35.4, M45.0-M45.9, N30.1, N70.92, O26.4, Q00-Q07, Q10-Q18, Q25.0-Q25.1, Q25.21, Q25.29, Q25.3, Q25.40-Q25.49, Q25.5-Q25.9, Q26-Q28, Q30-Q45, Q50, Q51.0-Q51.1, Q51.21-Q51.22, Q51.28, Q51.3-Q52.0, Q52.10-Q52.11, Q52.120-Q52.124, Q52.129, Q52.2-Q52.9, Q56.0, Q56.2-Q56.4, Q60-Q65, Q66.00-Q66.02, Q66.10-Q66.12, Q66.211-Q66.212, Q66.219, Q66.221-Q66.222, Q66.229, Q66.30-Q66.32, Q66.40-Q66.42, Q66.5-Q66.6, Q66.70-Q66.72, Q66.8, Q66.90-Q66.92, Q67-Q78, Q79.0-Q79.5, Q79.60-Q79.63, Q79.69, Q79.8-Q79.9, Q80-Q84, Q85.0-Q85.1, Q85.81-Q85.83, Q85.89, Q85.9, Q86, Q87.0, Q87.11, Q87.19, Q87.2-Q87.5, Q87.81-Q87.82, Q87.89, Q89-Q92, Q93.0-Q93.4, Q93.51, Q93.59, Q93.7-Q93.9, Q95-Q97, Q99 |
| **Current Tobacco Use** | F17, O99.33, Z72.0 |
| **Gastrointestinal Disease Crohn’s Disease** | K50-K52, K58, L88, O99.6 |
| **Gonococcal** | A54, O98.2 |
| **Group B Strep** | B95.1, J15.3, O99.82, Z22.330 |
| **HIV/AIDS** | B20, O98.7, Z21 |
| **Hepatitis** | A51.45, B00.81, B15-B19, B25.1, B26.81, B27, B58.1, B94.2, K70.1, K71.2-K71.6, K73, K75.2-K75.4, K75.81, K83.8, O98.4 |
| **Herpes Simplex** | A60.0-A60.1, B00 |
| **History Tobacco Use** | Z87.891 |
| **Hyperthyroidism Graves Disease** | E04-E05, E06.2 |
| **Mental Health** | F05-F06, F07.0, F07.89, F07.9, F09, F20-F25, F28-F31, F32.0-F32.5, F32.81, F32.89, F32.9, F32.A, F33, F34.0-F34.1, F34.81, F34.89, F34.9, F39-F41, F42.2-F42.4, F42.8-F42.9, F43, F44.0-F44.2, F44.4, F44.6-F44.9, F45, F48, F50.0, F50.2, F50.81-F50.82, F50.89, F50.9, F51-F55, F59-F60, F63-F66, F68-F69, G47.9, R45, R53.81, R53.83 |
| **Neuromuscular Disease** | O99.35 |
| **Obesity** | E66, O99.21, Z68.3-Z68.4 |
| **Other STI** | A49.3, A63-A64, B85.3, B97.7, N73.9, N74, O98.31 |
| **Polycystic Ovary Syndrome** | E28.2 |
| **Pre-existing Bleeding Disorder** | D66-D67, D68.00-D68.01, D68.020-D68.023, D68.029, D68.03-D68.04, D68.09, D68.1-D68.6, D69.8-D69.9, D75.821-D75.822, D75.828-D75.829, O72.3, O99.1, R58, R79.1, T45.51, T45.62, Z14.0 |
| **Pre-existing Hypertension** | I10-I13, I15, O10 |
| **Previous Cesarean** | O34.218-O34.219 |
| **Prior Fetal Death** | N96, O26.2 |
| **Pulmonary Hypertension** | I27.0, I27.20-I27.24, I27.29, I27.81, I27.9 |
| **Seizure Disorders** | F44.5, G40, R56 |
| **Sickle Cell** | D57 |
| **Substance Use** | F11.10-F11.15, F11.182, F11.188, F11.19, F11.2, F11.90-F11.95, F11.982, F11.988, F11.99, F12.1, F12.20-F12.22, F12.25, F12.28-F12.29, F12.90-F12.92, F12.95, F12.98-F12.99, F13.10-F13.12, F13.130-F13.132, F13.139, F13.14-F13.15, F13.180, F13.182, F13.188, F13.19-F13.25, F13.28-F13.29, F13.90-F13.92, F13.930-F13.931, F13.939, F13.94-F13.96, F13.980, F13.982, F13.988, F13.99, F14.10-F14.15, F14.180, F14.182, F14.188, F14.19-F14.25, F14.280, F14.282, F14.288, F14.29, F14.90-F14.95, F14.980, F14.982, F14.988, F14.99, F15.10-F15.15, F15.180, F15.182, F15.188, F15.19-F15.25, F15.280, F15.282, F15.288, F15.29, F15.90-F15.95, F15.980, F15.982, F15.988, F15.99, F16, F18.1, F18.20, F18.229, F18.24, F18.250, F18.29, F18.90, F18.929, F18.94, F18.951, F18.959, F18.97-F18.99, F19.10-F19.12, F19.130-F19.132, F19.139, F19.14-F19.17, F19.180, F19.182, F19.188, F19.19-F19.25, F19.27-F19.29, F19.90, F19.92-F19.99 |
| **Supervision of High Risk Pregnancy** | O09.0-O09.3, O09.5-O09.7, O09.82, O09.89, O09.9, O09.A |
| **Syphilis** | A50, A51.0-A51.3, A51.41-A51.44, A51.46, A51.49, A51.5, A51.9, A52.00-A52.02, A52.04-A52.06, A52.09, A52.1-A52.3, A52.7-A52.9, A53, A65, O98.1 |
| **Systemic Lupus Erythematosus** | M32 |
| **Trichomoniasis** | A59 |
| **Type I Pre-existing Diabetes** | E10.1-E10.2, E10.31, E10.3211-E10.3213, E10.3219, E10.3291-E10.3293, E10.3299, E10.3311-E10.3313, E10.3319, E10.3391-E10.3393, E10.3399, E10.3411-E10.3413, E10.3419, E10.3491-E10.3493, E10.3499, E10.3511-E10.3513, E10.3519, E10.352-E10.355, E10.3591-E10.3593, E10.3599, E10.36-E10.37, E10.39, E10.4-E10.6, E10.8-E10.9 |
| **Type II Pre-existing Diabetes** | E11.0, E11.10-E11.11, E11.2, E11.31, E11.3211-E11.3213, E11.3219, E11.3291-E11.3293, E11.3299, E11.3311-E11.3313, E11.3319, E11.3391-E11.3393, E11.3399, E11.3411-E11.3413, E11.3419, E11.3491-E11.3493, E11.3499, E11.3511-E11.3513, E11.3519, E11.352-E11.355, E11.3591-E11.3593, E11.3599, E11.36-E11.37, E11.39, E11.4-E11.6, E11.8-E11.9 |
| **Uterine Fibroids** | D25, O34.1 |
