# Supplementary material for: Association between COVID-19 by variant period and delivery-related outcomes in the United States
Source: PLoS One. 2026 Aug 12;21(8):e0355223. doi: 10.1371/journal.pone.0355223 (PMC13466025; doi:10.1371/journal.pone.0355223)
Supplement: Supplementary file 4 [file pone.0355223.s007.docx]

Supplemental Table 4. Adjusted Odds Ratios and Exponentiated Coefficients from Generalized Additive Models Incorporating Time-Series Splines to Assess Variant-Period Effects

| **Outcome** | **COVID Variant** | **Analysis Estimate** | **GAM Estimate** | **Estimate Difference** |
| --- | --- | --- | --- | --- |
| Mortality^1^ | Pre-Alpha | 13.11 (7.4-23.23) | 11.45 (6.23-21.05) | 1.66 |
| Mortality^1^ | Alpha | 13.03 (6.76-25.11) | 12.2 (6.29-23.67) | 0.83 |
| Mortality^1^ | Delta | 29.47 (19.57-44.39) | 29.36 (19.49-44.25) | 0.11 |
| Mortality^1^ | Omicron #1 | 3.55 (1.49-8.45) | 3.72 (1.56-8.89) | -0.17 |
| Mortality^1^ | Omicron #2 | 1.03 (0.12-8.86) | 1.17 (0.13-10.11) | -0.14 |
| 90-Day Mortality^1^ | Pre-Alpha | 10.09 (5.85-17.4) | 9.49 (5.36-16.81) | 0.6 |
| 90-Day Mortality^1^ | Alpha | 10.11 (5.39-18.94) | 9.8 (5.2-18.48) | 0.31 |
| 90-Day Mortality^1^ | Delta | 25.11 (17.34-36.34) | 25.06 (17.31-36.28) | 0.05 |
| 90-Day Mortality^1^ | Omicron #1 | 2.43 (1.03-5.73) | 2.48 (1.05-5.86) | -0.05 |
| 90-Day Mortality^1^ | Omicron #2 | 0.89 (0.12-6.85) | 0.94 (0.12-7.31) | -0.05 |
| SMM^1^ | Pre-Alpha | 5.55 (5.05-6.1) | 5.76 (5.22-6.35) | -0.21 |
| SMM^1^ | Alpha | 6.25 (5.57-7) | 6.45 (5.74-7.26) | -0.2 |
| SMM^1^ | Delta | 13 (12.06-14.01) | 12.97 (11.98-14.03) | 0.03 |
| SMM^1^ | Omicron #1 | 2.17 (1.93-2.43) | 2.18 (1.94-2.45) | -0.01 |
| SMM^1^ | Omicron #2 | 1.86 (1.55-2.24) | 1.78 (1.48-2.14) | 0.08 |
| 90-Day SMM^1^ | Pre-Alpha | 4.23 (3.87-4.62) | 4.34 (3.96-4.75) | -0.11 |
| 90-Day SMM^1^ | Alpha | 4.81 (4.32-5.35) | 4.88 (4.37-5.45) | -0.07 |
| 90-Day SMM^1^ | Delta | 9.64 (8.98-10.34) | 9.63 (8.94-10.38) | 0.01 |
| 90-Day SMM^1^ | Omicron #1 | 1.94 (1.75-2.14) | 1.99 (1.8-2.21) | -0.05 |
| 90-Day SMM^1^ | Omicron #2 | 1.59 (1.35-1.88) | 1.55 (1.31-1.83) | 0.04 |
| ICU Usage^1^ | Pre-Alpha | 5.88 (5.26-6.58) | 6.42 (5.72-7.21) | -0.54 |
| ICU Usage^1^ | Alpha | 4.28 (3.66-5) | 4.39 (3.74-5.16) | -0.11 |
| ICU Usage^1^ | Delta | 8.23 (7.42-9.13) | 8.01 (7.18-8.93) | 0.22 |
| ICU Usage^1^ | Omicron #1 | 1.74 (1.49-2.03) | 1.7 (1.45-1.99) | 0.04 |
| ICU Usage^1^ | Omicron #2 | 1.88 (1.48-2.4) | 1.84 (1.45-2.35) | 0.04 |
| Fetal Demise^1^ | Pre-Alpha | 1.53 (1.28-1.83) | 1.49 (1.24-1.78) | 0.04 |
| Fetal Demise^1^ | Alpha | 1.3 (1.02-1.67) | 1.29 (1-1.64) | 0.01 |
| Fetal Demise^1^ | Delta | 3.37 (2.95-3.84) | 3.37 (2.95-3.84) | 0 |
| Fetal Demise^1^ | Omicron #1 | 1.1 (0.92-1.3) | 1.11 (0.93-1.31) | -0.01 |
| Fetal Demise^1^ | Omicron #2 | 0.97 (0.73-1.29) | 1 (0.75-1.33) | -0.03 |
| Preterm Delivery^1^ | Pre-Alpha | 1.22 (1.12-1.32) | 1.17 (1.08-1.27) | 0.05 |
| Preterm Delivery^1^ | Alpha | 1.29 (1.16-1.43) | 1.28 (1.15-1.42) | 0.01 |
| Preterm Delivery^1^ | Delta | 1.42 (1.31-1.54) | 1.45 (1.33-1.57) | -0.03 |
| Preterm Delivery^1^ | Omicron #1 | 1.17 (1.09-1.25) | 1.2 (1.12-1.29) | -0.03 |
| Preterm Delivery^1^ | Omicron #2 | 1.36 (1.23-1.51) | 1.38 (1.25-1.53) | -0.02 |
| Cesarean Delivery^1^ | Pre-Alpha | 1.1 (1.05-1.15) | 1.1 (1.05-1.16) | 0 |
| Cesarean Delivery^1^ | Alpha | 1.08 (1.01-1.14) | 1.08 (1.01-1.14) | 0 |
| Cesarean Delivery^1^ | Delta | 1.2 (1.14-1.26) | 1.2 (1.14-1.26) | 0 |
| Cesarean Delivery^1^ | Omicron #1 | 1.05 (1.01-1.09) | 1.04 (1-1.08) | 0.01 |
| Cesarean Delivery^1^ | Omicron #2 | 1.16 (1.1-1.23) | 1.16 (1.09-1.23) | 0 |
| Cost^2^ | Pre-Alpha | 1.11 (1.1-1.12) | 1.11 (1.1-1.12) | 0 |
| Cost^2^ | Alpha | 1.09 (1.07-1.1) | 1.11 (1.09-1.12) | -0.02 |
| Cost^2^ | Delta | 1.17 (1.16-1.19) | 1.17 (1.16-1.19) | 0 |
| Cost^2^ | Omicron #1 | 1.05 (1.04-1.06) | 1.04 (1.03-1.05) | 0.01 |
| Cost^2^ | Omicron #2 | 1.08 (1.07-1.1) | 1.08 (1.06-1.09) | 0 |
| Length of Stay^2^ | Pre-Alpha | 1.04 (1.02-1.05) | 1.06 (1.04-1.07) | -0.02 |
| Length of Stay^2^ | Alpha | 1.06 (1.05-1.08) | 1.07 (1.05-1.08) | -0.01 |
| Length of Stay^2^ | Delta | 1.11 (1.1-1.13) | 1.11 (1.1-1.12) | 0 |
| Length of Stay^2^ | Omicron #1 | 1.02 (1.01-1.03) | 1.01 (1-1.02) | 0.01 |
| Length of Stay^2^ | Omicron #2 | 1.05 (1.04-1.07) | 1.03 (1.02-1.05) | 0.02 |
| ^1^ Odds Ratio  ^2^ Exponentiated Estimate | | | | |
