# Supplementary material for: Association between COVID-19 by variant period and delivery-related outcomes in the United States
Source: PLoS One. 2026 Aug 12;21(8):e0355223. doi: 10.1371/journal.pone.0355223 (PMC13466025; doi:10.1371/journal.pone.0355223)
Supplement: Supplementary file 5 [file pone.0355223.s008.docx]

Supplemental Table 5. Mortality Regression Coefficients

| **term** | **odds.ratio** | **p.value** | **conf.low** | **conf.high** |
| --- | --- | --- | --- | --- |
| (Intercept) | 0 | <.001 | 0 | 0 |
| COVID-19: Alpha | 13.028 | <.001 | 6.761 | 25.106 |
| COVID-19: Delta | 29.475 | <.001 | 19.569 | 44.394 |
| COVID-19: Omicron #1 | 3.553 | 0.004 | 1.493 | 8.451 |
| COVID-19: Omicron #2 | 1.033 | 0.976 | 0.121 | 8.858 |
| COVID-19: Pre-Alpha | 13.113 | <.001 | 7.401 | 23.234 |
| Age: 10-19 | 0.444 | 0.123 | 0.158 | 1.247 |
| Age: 20-24 | 0.626 | 0.069 | 0.378 | 1.037 |
| Age: 25-29 | 0.85 | 0.44 | 0.562 | 1.285 |
| Age: 35-39 | 1.67 | 0.012 | 1.118 | 2.495 |
| Age: 40-44 | 4.225 | <.001 | 2.652 | 6.731 |
| Age: 45-64 | 9.651 | <.001 | 3.367 | 27.662 |
| Race: American Indian | 0.586 | 0.525 | 0.113 | 3.045 |
| Race: Asian | 1.019 | 0.961 | 0.473 | 2.197 |
| Race: Black | 1.825 | 0.001 | 1.272 | 2.617 |
| Race: Other | 1.673 | 0.038 | 1.03 | 2.719 |
| Race: Pacific Islander | 0.788 | 0.816 | 0.107 | 5.819 |
| Race: Unable To Determine | 1.589 | 0.119 | 0.888 | 2.843 |
| Ethnicity: Hispanic Or Latino | 0.836 | 0.412 | 0.544 | 1.283 |
| Ethnicity: Unknown | 1.498 | 0.061 | 0.981 | 2.287 |
| Payor: Charity Indigent | 0 | 1 | 0 | - |
| Payor: Medicaid | 1.87 | <.001 | 1.352 | 2.585 |
| Payor: Medicare | 1.524 | 0.418 | 0.549 | 4.229 |
| Payor: Other Unknown | 1.293 | 0.478 | 0.636 | 2.631 |
| Admission Type: Information Not Available | 0.835 | 0.7 | 0.333 | 2.092 |
| Admission Type: Trauma Center/Emergency/Urgent | 1.7 | 0.001 | 1.247 | 2.317 |
| Admission Source: Clinic | 1.038 | 0.858 | 0.69 | 1.561 |
| Admission Source: Information Not Available/Other | 3.309 | 0.01 | 1.335 | 8.201 |
| Admission Source: Transfer | 4.137 | <.001 | 2.673 | 6.403 |
| Acquired Heart Disease | 17.058 | <.001 | 12.574 | 23.143 |
| Alcohol Use | 1.219 | 0.852 | 0.152 | 9.798 |
| Anemia | 1.05 | 0.796 | 0.727 | 1.516 |
| Asthma | 0.913 | 0.689 | 0.583 | 1.428 |
| Bariatric Surgery | 0.54 | 0.427 | 0.118 | 2.466 |
| Pre-existing Bleeding Disorder | 5.404 | <.001 | 3.906 | 7.476 |
| Cancer | 4.893 | <.001 | 2.594 | 9.231 |
| Cardiac Valvular Disease | 0.35 | 0.072 | 0.111 | 1.1 |
| Chlamydia | 0 | 1 | 0 | - |
| Chronic Ischemic Heart Disease | 1.038 | 0.961 | 0.24 | 4.487 |
| Chronic Renal Disease | 4.353 | <.001 | 2.551 | 7.429 |
| Congenital Heart Disease | 0 | 0.999 | 0 | - |
| Connective Tissue Or Autoimmune Disease Excluding Lupus | 1.127 | 0.636 | 0.688 | 1.845 |
| Current Tobacco Use | 1.274 | 0.375 | 0.747 | 2.172 |
| Gastrointestinal Disease Crohns Disease | 1.661 | 0.01 | 1.127 | 2.449 |
| Gonococcal | 0 | 1 | 0 | - |
| Group B Strep | 0.496 | 0.005 | 0.303 | 0.812 |
| Hepatitis | 1.58 | 0.278 | 0.692 | 3.612 |
| Herpes Simplex | 0.396 | 0.113 | 0.126 | 1.247 |
| History Tobacco Use | 0.736 | 0.266 | 0.428 | 1.264 |
| HIV/AIDS | 1.599 | 0.657 | 0.202 | 12.651 |
| Hyperthyroidism Graves Disease | 1.022 | 0.977 | 0.242 | 4.319 |
| Mental Health | 0.63 | 0.021 | 0.426 | 0.932 |
| Neuromuscular Disease | 5.699 | <.001 | 3.895 | 8.339 |
| Obesity | 1.217 | 0.232 | 0.882 | 1.68 |
| Other STI | 0 | 0.999 | 0 | - |
| Polycystic Ovary Syndrome | 0.65 | 0.554 | 0.155 | 2.716 |
| Pre-existing Hypertension | 1.09 | 0.707 | 0.696 | 1.705 |
| Previous Cesarean | 0.664 | 0.029 | 0.46 | 0.958 |
| Prior Fetal Death | 0 | 0.999 | 0 | - |
| Pulmonary Hypertension | 3.13 | 0.014 | 1.264 | 7.751 |
| Seizure Disorders | 1.648 | 0.087 | 0.93 | 2.923 |
| Sickle Cell | 1.081 | 0.874 | 0.413 | 2.829 |
| Substance Use | 1.189 | 0.578 | 0.646 | 2.186 |
| Supervision Of High Risk Pregnancy | 0.923 | 0.914 | 0.213 | 3.995 |
| Syphilis | 4.698 | 0.035 | 1.115 | 19.788 |
| Systemic Lupus Erythematosus | 0.758 | 0.723 | 0.165 | 3.496 |
| Trichomoniasis | 0 | 0.999 | 0 | - |
| Type I Pre-existing Diabetes | 0.408 | 0.43 | 0.044 | 3.793 |
| Type II Pre-existing Diabetes | 0.926 | 0.849 | 0.417 | 2.056 |
| Uterine Fibroids | 0.699 | 0.339 | 0.336 | 1.455 |

Supplemental Table 6. 90-Day Mortality Regression Coefficients

| **term** | **odds.ratio** | **p.value** | **conf.low** | **conf.high** |
| --- | --- | --- | --- | --- |
| (Intercept) | 0 | <.001 | 0 | 0 |
| COVID-19: Alpha | 10.106 | <.001 | 5.391 | 18.945 |
| COVID-19: Delta | 25.106 | <.001 | 17.345 | 36.339 |
| COVID-19: Omicron #1 | 2.431 | 0.042 | 1.032 | 5.726 |
| COVID-19: Omicron #2 | 0.89 | 0.911 | 0.115 | 6.853 |
| COVID-19: Pre-Alpha | 10.089 | <.001 | 5.85 | 17.4 |
| Age: 10-19 | 0.676 | 0.279 | 0.333 | 1.373 |
| Age: 20-24 | 0.598 | 0.018 | 0.391 | 0.914 |
| Age: 25-29 | 0.854 | 0.367 | 0.607 | 1.203 |
| Age: 35-39 | 1.466 | 0.03 | 1.038 | 2.071 |
| Age: 40-44 | 3.322 | <.001 | 2.193 | 5.031 |
| Age: 45-64 | 6.628 | <.001 | 2.364 | 18.579 |
| Race: American Indian | 1.697 | 0.25 | 0.69 | 4.174 |
| Race: Asian | 1.062 | 0.86 | 0.545 | 2.068 |
| Race: Black | 1.656 | 0.002 | 1.212 | 2.261 |
| Race: Other | 1.523 | 0.053 | 0.995 | 2.332 |
| Race: Pacific Islander | 1.126 | 0.869 | 0.274 | 4.625 |
| Race: Unable To Determine | 1.546 | 0.09 | 0.934 | 2.558 |
| Ethnicity: Hispanic Or Latino | 0.752 | 0.14 | 0.516 | 1.098 |
| Ethnicity: Unknown | 1.621 | 0.006 | 1.147 | 2.292 |
| Payor: Charity Indigent | 0 | 0.999 | 0 | - |
| Payor: Medicaid | 2.2 | <.001 | 1.651 | 2.93 |
| Payor: Medicare | 1.784 | 0.187 | 0.756 | 4.212 |
| Payor: Other Unknown | 1.708 | 0.068 | 0.96 | 3.037 |
| Admission Type: Information Not Available | 1.004 | 0.991 | 0.505 | 1.999 |
| Admission Type: Trauma Center/Emergency/Urgent | 1.421 | 0.007 | 1.099 | 1.838 |
| Admission Source: Clinic | 1.024 | 0.893 | 0.727 | 1.441 |
| Admission Source: Information Not Available/Other | 2.84 | 0.008 | 1.321 | 6.104 |
| Admission Source: Transfer | 3.257 | <.001 | 2.166 | 4.898 |
| Acquired Heart Disease | 10.845 | <.001 | 8.38 | 14.034 |
| Alcohol Use | 1.449 | 0.624 | 0.328 | 6.397 |
| Anemia | 0.999 | 0.997 | 0.724 | 1.379 |
| Asthma | 1.109 | 0.577 | 0.771 | 1.596 |
| Bariatric Surgery | 0.732 | 0.613 | 0.219 | 2.448 |
| Pre-existing Bleeding Disorder | 4.549 | <.001 | 3.383 | 6.118 |
| Cancer | 4.284 | <.001 | 2.375 | 7.725 |
| Cardiac Valvular Disease | 0.641 | 0.318 | 0.268 | 1.533 |
| Chlamydia | 1.158 | 0.885 | 0.159 | 8.412 |
| Chronic Ischemic Heart Disease | 1.219 | 0.748 | 0.364 | 4.083 |
| Chronic Renal Disease | 4.826 | <.001 | 2.989 | 7.793 |
| Congenital Heart Disease | 0 | 0.998 | 0 | - |
| Connective Tissue Or Autoimmune Disease Excluding Lupus | 1.136 | 0.575 | 0.728 | 1.773 |
| Current Tobacco Use | 1.585 | 0.028 | 1.051 | 2.389 |
| Gastrointestinal Disease Crohns Disease | 1.628 | 0.006 | 1.153 | 2.3 |
| Gonococcal | 0 | 0.999 | 0 | - |
| Group B Strep | 0.549 | 0.003 | 0.37 | 0.816 |
| Hepatitis | 1.61 | 0.151 | 0.841 | 3.082 |
| Herpes Simplex | 0.349 | 0.038 | 0.129 | 0.943 |
| History Tobacco Use | 0.767 | 0.258 | 0.484 | 1.214 |
| HIV/AIDS | 2.676 | 0.184 | 0.627 | 11.425 |
| Hyperthyroidism Graves Disease | 1.264 | 0.693 | 0.395 | 4.046 |
| Mental Health | 0.842 | 0.296 | 0.611 | 1.162 |
| Neuromuscular Disease | 4.649 | <.001 | 3.293 | 6.563 |
| Obesity | 1.242 | 0.128 | 0.939 | 1.642 |
| Other STI | 0.377 | 0.344 | 0.05 | 2.842 |
| Polycystic Ovary Syndrome | 0.507 | 0.346 | 0.124 | 2.078 |
| Pre-existing Hypertension | 1.141 | 0.509 | 0.771 | 1.69 |
| Previous Cesarean | 0.718 | 0.036 | 0.527 | 0.979 |
| Prior Fetal Death | 0 | 0.998 | 0 | - |
| Pulmonary Hypertension | 2.651 | 0.022 | 1.149 | 6.118 |
| Seizure Disorders | 1.381 | 0.242 | 0.804 | 2.372 |
| Sickle Cell | 1.718 | 0.14 | 0.837 | 3.528 |
| Substance Use | 1.589 | 0.044 | 1.012 | 2.495 |
| Supervision Of High Risk Pregnancy | 0.684 | 0.602 | 0.164 | 2.855 |
| Syphilis | 2.236 | 0.274 | 0.529 | 9.443 |
| Systemic Lupus Erythematosus | 0.66 | 0.582 | 0.151 | 2.892 |
| Trichomoniasis | 0.438 | 0.423 | 0.058 | 3.299 |
| Type I Pre-existing Diabetes | 0.351 | 0.342 | 0.041 | 3.04 |
| Type II Pre-existing Diabetes | 1.123 | 0.739 | 0.568 | 2.221 |
| Uterine Fibroids | 0.822 | 0.554 | 0.429 | 1.575 |

Supplemental Table 7. SMM Regression Coefficients

| **term** | **odds.ratio** | **p.value** | **conf.low** | **conf.high** |
| --- | --- | --- | --- | --- |
| (Intercept) | 0.003 | <.001 | 0.002 | 0.004 |
| COVID-19: Alpha | 6.247 | <.001 | 5.573 | 7.002 |
| COVID-19: Delta | 13.002 | <.001 | 12.062 | 14.014 |
| COVID-19: Omicron #1 | 2.166 | <.001 | 1.933 | 2.426 |
| COVID-19: Omicron #2 | 1.861 | <.001 | 1.55 | 2.236 |
| COVID-19: Pre-Alpha | 5.549 | <.001 | 5.047 | 6.102 |
| Age: 10-19 | 1.037 | 0.339 | 0.963 | 1.117 |
| Age: 20-24 | 0.838 | <.001 | 0.801 | 0.877 |
| Age: 25-29 | 0.851 | <.001 | 0.819 | 0.884 |
| Age: 35-39 | 1.256 | <.001 | 1.208 | 1.307 |
| Age: 40-44 | 1.678 | <.001 | 1.58 | 1.782 |
| Age: 45-64 | 2.245 | <.001 | 1.88 | 2.68 |
| Race: American Indian | 1.399 | <.001 | 1.231 | 1.59 |
| Race: Asian | 1.368 | <.001 | 1.281 | 1.46 |
| Race: Black | 1.431 | <.001 | 1.377 | 1.487 |
| Race: Other | 1.165 | <.001 | 1.106 | 1.227 |
| Race: Pacific Islander | 1.856 | <.001 | 1.631 | 2.111 |
| Race: Unable To Determine | 1.267 | <.001 | 1.191 | 1.349 |
| Ethnicity: Hispanic Or Latino | 1.06 | 0.005 | 1.018 | 1.105 |
| Ethnicity: Unknown | 1.052 | 0.037 | 1.003 | 1.103 |
| Payor: Charity Indigent | 0.813 | 0.447 | 0.476 | 1.387 |
| Payor: Medicaid | 1.077 | <.001 | 1.043 | 1.112 |
| Payor: Medicare | 1.302 | <.001 | 1.149 | 1.475 |
| Payor: Other Unknown | 0.951 | 0.154 | 0.887 | 1.019 |
| Admission Type: Information Not Available | 0.969 | 0.454 | 0.893 | 1.052 |
| Admission Type: Trauma Center/Emergency/Urgent | 1.432 | <.001 | 1.391 | 1.475 |
| Admission Source: Clinic | 0.959 | 0.032 | 0.924 | 0.996 |
| Admission Source: Information Not Available/Other | 1.264 | <.001 | 1.116 | 1.431 |
| Admission Source: Transfer | 3.149 | <.001 | 2.948 | 3.363 |
| Acquired Heart Disease | 4.212 | <.001 | 4.065 | 4.365 |
| Alcohol Use | 1.424 | 0.01 | 1.088 | 1.863 |
| Anemia | 1.274 | <.001 | 1.227 | 1.323 |
| Asthma | 1.104 | <.001 | 1.055 | 1.157 |
| Bariatric Surgery | 0.767 | <.001 | 0.663 | 0.887 |
| Pre-existing Bleeding Disorder | 4.819 | <.001 | 4.628 | 5.018 |
| Cancer | 1.546 | <.001 | 1.357 | 1.761 |
| Cardiac Valvular Disease | 2.294 | <.001 | 2.023 | 2.602 |
| Chlamydia | 1.222 | 0.081 | 0.975 | 1.53 |
| Chronic Ischemic Heart Disease | 1.597 | <.001 | 1.261 | 2.023 |
| Chronic Renal Disease | 26.719 | <.001 | 25.045 | 28.506 |
| Congenital Heart Disease | 1.367 | 0.012 | 1.072 | 1.743 |
| Connective Tissue Or Autoimmune Disease Excluding Lupus | 1.003 | 0.933 | 0.941 | 1.069 |
| Current Tobacco Use | 1.232 | <.001 | 1.163 | 1.307 |
| Gastrointestinal Disease Crohns Disease | 1.882 | <.001 | 1.8 | 1.967 |
| Gonococcal | 0.98 | 0.93 | 0.624 | 1.54 |
| Group B Strep | 0.716 | <.001 | 0.688 | 0.745 |
| Hepatitis | 1.436 | <.001 | 1.288 | 1.601 |
| Herpes Simplex | 0.832 | <.001 | 0.772 | 0.897 |
| History Tobacco Use | 0.99 | 0.704 | 0.941 | 1.042 |
| HIV/AIDS | 1.128 | 0.413 | 0.846 | 1.505 |
| Hyperthyroidism Graves Disease | 1.263 | 0.003 | 1.085 | 1.471 |
| Mental Health | 1.247 | <.001 | 1.201 | 1.295 |
| Neuromuscular Disease | 1.618 | <.001 | 1.516 | 1.726 |
| Obesity | 1.338 | <.001 | 1.294 | 1.384 |
| Other STI | 0.891 | 0.228 | 0.738 | 1.075 |
| Polycystic Ovary Syndrome | 1.056 | 0.35 | 0.942 | 1.183 |
| Pre-existing Hypertension | 1.319 | <.001 | 1.254 | 1.389 |
| Previous Cesarean | 1.389 | <.001 | 1.344 | 1.435 |
| Prior Fetal Death | 1.098 | 0.34 | 0.906 | 1.329 |
| Pulmonary Hypertension | 6.687 | <.001 | 5.462 | 8.188 |
| Seizure Disorders | 3.505 | <.001 | 3.197 | 3.842 |
| Sickle Cell | 1.274 | <.001 | 1.141 | 1.423 |
| Substance Use | 1.606 | <.001 | 1.508 | 1.711 |
| Supervision Of High Risk Pregnancy | 1.109 | 0.21 | 0.943 | 1.303 |
| Syphilis | 1.582 | <.001 | 1.275 | 1.962 |
| Systemic Lupus Erythematosus | 0.792 | 0.02 | 0.651 | 0.964 |
| Trichomoniasis | 1.28 | 0.023 | 1.034 | 1.584 |
| Type I Pre-existing Diabetes | 2.418 | <.001 | 2.102 | 2.781 |
| Type II Pre-existing Diabetes | 1.381 | <.001 | 1.247 | 1.529 |
| Uterine Fibroids | 1.372 | <.001 | 1.278 | 1.472 |

Supplemental Table 8. 90-Day SMM Regression Coefficients

| **term** | **odds.ratio** | **p.value** | **conf.low** | **conf.high** |
| --- | --- | --- | --- | --- |
| (Intercept) | 0.005 | <.001 | 0.004 | 0.005 |
| COVID-19: Alpha | 4.811 | <.001 | 4.323 | 5.353 |
| COVID-19: Delta | 9.638 | <.001 | 8.981 | 10.342 |
| COVID-19: Omicron #1 | 1.935 | <.001 | 1.751 | 2.139 |
| COVID-19: Omicron #2 | 1.592 | <.001 | 1.348 | 1.879 |
| COVID-19: Pre-Alpha | 4.228 | <.001 | 3.867 | 4.622 |
| Age: 10-19 | 1.051 | 0.115 | 0.988 | 1.118 |
| Age: 20-24 | 0.875 | <.001 | 0.843 | 0.908 |
| Age: 25-29 | 0.876 | <.001 | 0.849 | 0.904 |
| Age: 35-39 | 1.245 | <.001 | 1.205 | 1.288 |
| Age: 40-44 | 1.608 | <.001 | 1.526 | 1.693 |
| Age: 45-64 | 2.033 | <.001 | 1.734 | 2.383 |
| Race: American Indian | 1.308 | <.001 | 1.172 | 1.46 |
| Race: Asian | 1.286 | <.001 | 1.216 | 1.361 |
| Race: Black | 1.523 | <.001 | 1.475 | 1.572 |
| Race: Other | 1.109 | <.001 | 1.061 | 1.16 |
| Race: Pacific Islander | 1.618 | <.001 | 1.445 | 1.811 |
| Race: Unable To Determine | 1.181 | <.001 | 1.12 | 1.246 |
| Ethnicity: Hispanic Or Latino | 1.05 | 0.006 | 1.014 | 1.088 |
| Ethnicity: Unknown | 1.026 | 0.21 | 0.986 | 1.068 |
| Payor: Charity Indigent | 0.818 | 0.376 | 0.524 | 1.277 |
| Payor: Medicaid | 1.09 | <.001 | 1.061 | 1.119 |
| Payor: Medicare | 1.365 | <.001 | 1.227 | 1.519 |
| Payor: Other Unknown | 0.943 | 0.048 | 0.89 | 0.999 |
| Admission Type: Information Not Available | 1.014 | 0.679 | 0.95 | 1.082 |
| Admission Type: Trauma Center/Emergency/Urgent | 1.302 | <.001 | 1.27 | 1.333 |
| Admission Source: Clinic | 0.978 | 0.174 | 0.948 | 1.01 |
| Admission Source: Information Not Available/Other | 1.177 | 0.002 | 1.061 | 1.306 |
| Admission Source: Transfer | 2.644 | <.001 | 2.49 | 2.808 |
| Acquired Heart Disease | 3.338 | <.001 | 3.233 | 3.447 |
| Alcohol Use | 1.352 | 0.014 | 1.063 | 1.719 |
| Anemia | 1.247 | <.001 | 1.208 | 1.288 |
| Asthma | 1.145 | <.001 | 1.101 | 1.19 |
| Bariatric Surgery | 0.78 | <.001 | 0.689 | 0.884 |
| Pre-existing Bleeding Disorder | 3.951 | <.001 | 3.809 | 4.099 |
| Cancer | 1.744 | <.001 | 1.565 | 1.943 |
| Cardiac Valvular Disease | 2.204 | <.001 | 1.957 | 2.481 |
| Chlamydia | 1.011 | 0.918 | 0.823 | 1.242 |
| Chronic Ischemic Heart Disease | 1.826 | <.001 | 1.479 | 2.254 |
| Chronic Renal Disease | 19.519 | <.001 | 18.354 | 20.757 |
| Congenital Heart Disease | 1.401 | 0.003 | 1.125 | 1.745 |
| Connective Tissue Or Autoimmune Disease Excluding Lupus | 1.032 | 0.266 | 0.976 | 1.091 |
| Current Tobacco Use | 1.318 | <.001 | 1.256 | 1.383 |
| Gastrointestinal Disease Crohns Disease | 1.703 | <.001 | 1.637 | 1.771 |
| Gonococcal | 0.959 | 0.836 | 0.646 | 1.424 |
| Group B Strep | 0.775 | <.001 | 0.751 | 0.801 |
| Hepatitis | 1.376 | <.001 | 1.25 | 1.515 |
| Herpes Simplex | 0.872 | <.001 | 0.819 | 0.928 |
| History Tobacco Use | 1.036 | 0.108 | 0.992 | 1.081 |
| HIV/AIDS | 1.09 | 0.508 | 0.845 | 1.405 |
| Hyperthyroidism Graves Disease | 1.257 | 0.001 | 1.102 | 1.434 |
| Mental Health | 1.238 | <.001 | 1.198 | 1.278 |
| Neuromuscular Disease | 1.545 | <.001 | 1.459 | 1.636 |
| Obesity | 1.373 | <.001 | 1.335 | 1.412 |
| Other STI | 0.997 | 0.97 | 0.855 | 1.163 |
| Polycystic Ovary Syndrome | 1.067 | 0.189 | 0.969 | 1.175 |
| Pre-existing Hypertension | 1.368 | <.001 | 1.31 | 1.429 |
| Previous Cesarean | 1.336 | <.001 | 1.3 | 1.373 |
| Prior Fetal Death | 1.094 | 0.291 | 0.926 | 1.292 |
| Pulmonary Hypertension | 6.101 | <.001 | 5.018 | 7.418 |
| Seizure Disorders | 2.875 | <.001 | 2.644 | 3.127 |
| Sickle Cell | 1.347 | <.001 | 1.23 | 1.475 |
| Substance Use | 1.483 | <.001 | 1.405 | 1.566 |
| Supervision Of High Risk Pregnancy | 1.065 | 0.384 | 0.925 | 1.226 |
| Syphilis | 1.428 | <.001 | 1.177 | 1.732 |
| Systemic Lupus Erythematosus | 0.976 | 0.776 | 0.826 | 1.154 |
| Trichomoniasis | 1.319 | 0.003 | 1.1 | 1.582 |
| Type I Pre-existing Diabetes | 2.527 | <.001 | 2.236 | 2.856 |
| Type II Pre-existing Diabetes | 1.468 | <.001 | 1.345 | 1.602 |
| Uterine Fibroids | 1.343 | <.001 | 1.264 | 1.428 |

Supplemental Table 9. ICU Usage Regression Coefficients

| **term** | **odds.ratio** | **p.value** | **conf.low** | **conf.high** |
| --- | --- | --- | --- | --- |
| (Intercept) | 0.001 | <.001 | 0.001 | 0.002 |
| COVID-19: Alpha | 4.275 | <.001 | 3.656 | 4.999 |
| COVID-19: Delta | 8.231 | <.001 | 7.421 | 9.129 |
| COVID-19: Omicron #1 | 1.741 | <.001 | 1.494 | 2.028 |
| COVID-19: Omicron #2 | 1.884 | <.001 | 1.481 | 2.395 |
| COVID-19: Pre-Alpha | 5.88 | <.001 | 5.257 | 6.578 |
| Age: 10-19 | 1.07 | 0.134 | 0.979 | 1.168 |
| Age: 20-24 | 0.915 | 0.001 | 0.868 | 0.965 |
| Age: 25-29 | 0.871 | <.001 | 0.832 | 0.912 |
| Age: 35-39 | 1.175 | <.001 | 1.118 | 1.235 |
| Age: 40-44 | 1.531 | <.001 | 1.418 | 1.654 |
| Age: 45-64 | 1.948 | <.001 | 1.546 | 2.455 |
| Race: American Indian | 1.83 | <.001 | 1.606 | 2.086 |
| Race: Asian | 1.423 | <.001 | 1.308 | 1.548 |
| Race: Black | 1.409 | <.001 | 1.345 | 1.477 |
| Race: Other | 0.676 | <.001 | 0.623 | 0.734 |
| Race: Pacific Islander | 1.768 | <.001 | 1.44 | 2.17 |
| Race: Unable To Determine | 2.173 | <.001 | 2.044 | 2.31 |
| Ethnicity: Hispanic Or Latino | 0.856 | <.001 | 0.814 | 0.899 |
| Ethnicity: Unknown | 0.91 | 0.004 | 0.853 | 0.97 |
| Payor: Charity Indigent | 0.826 | 0.574 | 0.424 | 1.609 |
| Payor: Medicaid | 0.933 | <.001 | 0.897 | 0.97 |
| Payor: Medicare | 0.783 | 0.006 | 0.656 | 0.933 |
| Payor: Other Unknown | 0.964 | 0.386 | 0.888 | 1.047 |
| Admission Type: Information Not Available | 1.274 | <.001 | 1.176 | 1.379 |
| Admission Type: Trauma Center/Emergency/Urgent | 1.013 | 0.481 | 0.977 | 1.05 |
| Admission Source: Clinic | 0.732 | <.001 | 0.696 | 0.768 |
| Admission Source: Information Not Available/Other | 1.832 | <.001 | 1.622 | 2.07 |
| Admission Source: Transfer | 4.019 | <.001 | 3.744 | 4.315 |
| Acquired Heart Disease | 3.466 | <.001 | 3.314 | 3.625 |
| Alcohol Use | 1.769 | <.001 | 1.304 | 2.4 |
| Anemia | 1.185 | <.001 | 1.129 | 1.244 |
| Asthma | 1.125 | <.001 | 1.064 | 1.191 |
| Bariatric Surgery | 0.869 | 0.105 | 0.734 | 1.029 |
| Pre-existing Bleeding Disorder | 3.751 | <.001 | 3.553 | 3.96 |
| Cancer | 1.502 | <.001 | 1.276 | 1.767 |
| Cardiac Valvular Disease | 2.064 | <.001 | 1.761 | 2.42 |
| Chlamydia | 1.349 | 0.028 | 1.033 | 1.761 |
| Chronic Ischemic Heart Disease | 1.424 | 0.016 | 1.067 | 1.902 |
| Chronic Renal Disease | 4.84 | <.001 | 4.331 | 5.407 |
| Congenital Heart Disease | 2.27 | <.001 | 1.774 | 2.905 |
| Connective Tissue Or Autoimmune Disease Excluding Lupus | 1.027 | 0.504 | 0.949 | 1.112 |
| Current Tobacco Use | 1.337 | <.001 | 1.247 | 1.434 |
| Gastrointestinal Disease Crohns Disease | 1.858 | <.001 | 1.763 | 1.958 |
| Gonococcal | 1.112 | 0.683 | 0.668 | 1.853 |
| Group B Strep | 0.689 | <.001 | 0.656 | 0.724 |
| Hepatitis | 1.279 | 0.001 | 1.113 | 1.47 |
| Herpes Simplex | 0.845 | <.001 | 0.77 | 0.926 |
| History Tobacco Use | 0.975 | 0.427 | 0.916 | 1.038 |
| HIV/AIDS | 1.127 | 0.501 | 0.795 | 1.598 |
| Hyperthyroidism Graves Disease | 0.973 | 0.795 | 0.788 | 1.2 |
| Mental Health | 1.438 | <.001 | 1.375 | 1.503 |
| Neuromuscular Disease | 1.716 | <.001 | 1.59 | 1.853 |
| Obesity | 1.574 | <.001 | 1.513 | 1.637 |
| Other STI | 0.73 | 0.016 | 0.565 | 0.944 |
| Polycystic Ovary Syndrome | 1.225 | 0.002 | 1.078 | 1.392 |
| Pre-existing Hypertension | 1.571 | <.001 | 1.482 | 1.665 |
| Previous Cesarean | 1.01 | 0.652 | 0.968 | 1.053 |
| Prior Fetal Death | 0.584 | <.001 | 0.433 | 0.788 |
| Pulmonary Hypertension | 6.368 | <.001 | 5.078 | 7.986 |
| Seizure Disorders | 2.474 | <.001 | 2.205 | 2.776 |
| Sickle Cell | 1.18 | 0.025 | 1.021 | 1.364 |
| Substance Use | 1.669 | <.001 | 1.543 | 1.804 |
| Supervision Of High Risk Pregnancy | 0.981 | 0.849 | 0.805 | 1.196 |
| Syphilis | 1.355 | 0.027 | 1.035 | 1.773 |
| Systemic Lupus Erythematosus | 0.832 | 0.146 | 0.648 | 1.067 |
| Trichomoniasis | 1.092 | 0.51 | 0.84 | 1.419 |
| Type I Pre-existing Diabetes | 3.851 | <.001 | 3.318 | 4.468 |
| Type II Pre-existing Diabetes | 1.898 | <.001 | 1.697 | 2.123 |
| Uterine Fibroids | 1.332 | <.001 | 1.218 | 1.458 |

Supplemental Table 10. Fetal Death Regression Coefficients

| **term** | **odds.ratio** | **p.value** | **conf.low** | **conf.high** |
| --- | --- | --- | --- | --- |
| (Intercept) | 0.004 | <.001 | 0.004 | 0.005 |
| COVID-19: Alpha | 1.304 | 0.034 | 1.02 | 1.668 |
| COVID-19: Delta | 3.366 | <.001 | 2.951 | 3.84 |
| COVID-19: Omicron #1 | 1.096 | 0.29 | 0.925 | 1.299 |
| COVID-19: Omicron #2 | 0.973 | 0.85 | 0.731 | 1.294 |
| COVID-19: Pre-Alpha | 1.529 | <.001 | 1.28 | 1.827 |
| Age: 10-19 | 1.101 | 0.013 | 1.021 | 1.188 |
| Age: 20-24 | 0.971 | 0.231 | 0.926 | 1.019 |
| Age: 25-29 | 0.967 | 0.116 | 0.927 | 1.008 |
| Age: 35-39 | 1.176 | <.001 | 1.122 | 1.233 |
| Age: 40-44 | 1.661 | <.001 | 1.545 | 1.785 |
| Age: 45-64 | 2.065 | <.001 | 1.643 | 2.595 |
| Race: American Indian | 1.177 | 0.04 | 1.008 | 1.375 |
| Race: Asian | 0.876 | 0.003 | 0.802 | 0.956 |
| Race: Black | 2.043 | <.001 | 1.962 | 2.126 |
| Race: Other | 1.197 | <.001 | 1.128 | 1.269 |
| Race: Pacific Islander | 1.582 | <.001 | 1.367 | 1.832 |
| Race: Unable To Determine | 1.189 | <.001 | 1.107 | 1.277 |
| Ethnicity: Hispanic Or Latino | 0.934 | 0.005 | 0.892 | 0.979 |
| Ethnicity: Unknown | 1.06 | 0.028 | 1.006 | 1.117 |
| Payor: Charity Indigent | 2.116 | <.001 | 1.488 | 3.009 |
| Payor: Medicaid | 1.225 | <.001 | 1.182 | 1.269 |
| Payor: Medicare | 1.369 | <.001 | 1.165 | 1.608 |
| Payor: Other Unknown | 1.49 | <.001 | 1.397 | 1.589 |
| Admission Type: Information Not Available | 1.264 | <.001 | 1.167 | 1.369 |
| Admission Type: Trauma Center/Emergency/Urgent | 1.554 | <.001 | 1.504 | 1.606 |
| Admission Source: Clinic | 0.978 | 0.307 | 0.937 | 1.021 |
| Admission Source: Information Not Available/Other | 1.341 | <.001 | 1.181 | 1.523 |
| Admission Source: Transfer | 2.164 | <.001 | 1.986 | 2.357 |
| Acquired Heart Disease | 1.133 | <.001 | 1.063 | 1.209 |
| Alcohol Use | 1.725 | 0.001 | 1.268 | 2.346 |
| Anemia | 0.669 | <.001 | 0.633 | 0.707 |
| Asthma | 0.906 | 0.002 | 0.852 | 0.963 |
| Bariatric Surgery | 1.147 | 0.127 | 0.962 | 1.367 |
| Pre-existing Bleeding Disorder | 1.365 | <.001 | 1.265 | 1.472 |
| Cancer | 1.06 | 0.576 | 0.865 | 1.298 |
| Cardiac Valvular Disease | 0.86 | 0.395 | 0.607 | 1.217 |
| Chlamydia | 0.989 | 0.932 | 0.764 | 1.28 |
| Chronic Ischemic Heart Disease | 1.247 | 0.33 | 0.8 | 1.946 |
| Chronic Renal Disease | 3.171 | <.001 | 2.77 | 3.629 |
| Congenital Heart Disease | 1.203 | 0.406 | 0.778 | 1.859 |
| Connective Tissue Or Autoimmune Disease Excluding Lupus | 1.281 | <.001 | 1.179 | 1.392 |
| Current Tobacco Use | 1.45 | <.001 | 1.362 | 1.543 |
| Gastrointestinal Disease Crohns Disease | 0.833 | <.001 | 0.772 | 0.899 |
| Gonococcal | 1.481 | 0.053 | 0.996 | 2.204 |
| Group B Strep | 0.24 | <.001 | 0.224 | 0.257 |
| Hepatitis | 0.977 | 0.763 | 0.839 | 1.138 |
| Herpes Simplex | 0.525 | <.001 | 0.471 | 0.585 |
| History Tobacco Use | 0.966 | 0.269 | 0.908 | 1.027 |
| HIV/AIDS | 0.87 | 0.504 | 0.578 | 1.309 |
| Hyperthyroidism Graves Disease | 1.185 | 0.099 | 0.968 | 1.451 |
| Mental Health | 1.254 | <.001 | 1.198 | 1.313 |
| Neuromuscular Disease | 0.989 | 0.842 | 0.89 | 1.1 |
| Obesity | 0.873 | <.001 | 0.836 | 0.912 |
| Other STI | 0.92 | 0.479 | 0.73 | 1.16 |
| Polycystic Ovary Syndrome | 1.329 | <.001 | 1.159 | 1.523 |
| Pre-existing Hypertension | 1.453 | <.001 | 1.364 | 1.548 |
| Previous Cesarean | 0.865 | <.001 | 0.83 | 0.902 |
| Prior Fetal Death | 3.268 | <.001 | 2.794 | 3.822 |
| Pulmonary Hypertension | 0.99 | 0.97 | 0.567 | 1.726 |
| Seizure Disorders | 1.178 | 0.074 | 0.984 | 1.41 |
| Sickle Cell | 0.926 | 0.298 | 0.801 | 1.07 |
| Substance Use | 1.501 | <.001 | 1.398 | 1.613 |
| Supervision Of High Risk Pregnancy | 1.459 | <.001 | 1.229 | 1.732 |
| Syphilis | 2.958 | <.001 | 2.461 | 3.557 |
| Systemic Lupus Erythematosus | 1.668 | <.001 | 1.313 | 2.12 |
| Trichomoniasis | 1.54 | <.001 | 1.231 | 1.927 |
| Type I Pre-existing Diabetes | 3.455 | <.001 | 2.946 | 4.051 |
| Type II Pre-existing Diabetes | 2.709 | <.001 | 2.433 | 3.016 |
| Uterine Fibroids | 0.982 | 0.718 | 0.888 | 1.085 |

Supplemental Table 11. Preterm Delivery Regression Coefficients

| **term** | **odds.ratio** | **p.value** | **conf.low** | **conf.high** |
| --- | --- | --- | --- | --- |
| (Intercept) | 0.022 | <.001 | 0.019 | 0.026 |
| COVID-19: Alpha | 1.286 | <.001 | 1.159 | 1.427 |
| COVID-19: Delta | 1.42 | <.001 | 1.306 | 1.543 |
| COVID-19: Omicron #1 | 1.171 | <.001 | 1.094 | 1.254 |
| COVID-19: Omicron #2 | 1.361 | <.001 | 1.229 | 1.507 |
| COVID-19: Pre-Alpha | 1.216 | <.001 | 1.119 | 1.321 |
| Age: 10-19 | 1.202 | <.001 | 1.166 | 1.24 |
| Age: 20-24 | 1.071 | <.001 | 1.05 | 1.092 |
| Age: 25-29 | 1.003 | 0.721 | 0.986 | 1.021 |
| Age: 35-39 | 1.04 | <.001 | 1.019 | 1.062 |
| Age: 40-44 | 1.204 | <.001 | 1.162 | 1.247 |
| Age: 45-64 | 1.263 | <.001 | 1.116 | 1.429 |
| Race: American Indian | 1.201 | <.001 | 1.13 | 1.277 |
| Race: Asian | 1.036 | 0.04 | 1.002 | 1.071 |
| Race: Black | 1.438 | <.001 | 1.413 | 1.464 |
| Race: Other | 1.055 | <.001 | 1.03 | 1.081 |
| Race: Pacific Islander | 1.386 | <.001 | 1.304 | 1.474 |
| Race: Unable To Determine | 1.047 | 0.003 | 1.016 | 1.079 |
| Ethnicity: Hispanic Or Latino | 1.098 | <.001 | 1.077 | 1.119 |
| Ethnicity: Unknown | 1.052 | <.001 | 1.029 | 1.076 |
| Payor: Charity Indigent | 1.409 | <.001 | 1.178 | 1.686 |
| Payor: Medicaid | 1.288 | <.001 | 1.269 | 1.307 |
| Payor: Medicare | 1.407 | <.001 | 1.308 | 1.512 |
| Payor: Other Unknown | 1.086 | <.001 | 1.053 | 1.12 |
| Admission Type: Information Not Available | 1.403 | <.001 | 1.356 | 1.451 |
| Admission Type: Trauma Center/Emergency/Urgent | 1.92 | <.001 | 1.894 | 1.947 |
| Admission Source: Clinic | 0.89 | <.001 | 0.874 | 0.907 |
| Admission Source: Information Not Available/Other | 1.32 | <.001 | 1.248 | 1.395 |
| Admission Source: Transfer | 3.374 | <.001 | 3.263 | 3.489 |
| Acquired Heart Disease | 1.137 | <.001 | 1.106 | 1.169 |
| Alcohol Use | 1.29 | 0.001 | 1.106 | 1.503 |
| Anemia | 1.024 | 0.02 | 1.004 | 1.045 |
| Asthma | 1.065 | <.001 | 1.04 | 1.091 |
| Bariatric Surgery | 1.335 | <.001 | 1.244 | 1.433 |
| Pre-existing Bleeding Disorder | 1.111 | <.001 | 1.072 | 1.15 |
| Cancer | 1.161 | 0.001 | 1.067 | 1.263 |
| Cardiac Valvular Disease | 1.101 | 0.18 | 0.956 | 1.268 |
| Chlamydia | 1.522 | <.001 | 1.39 | 1.666 |
| Chronic Ischemic Heart Disease | 1.095 | 0.434 | 0.872 | 1.376 |
| Chronic Renal Disease | 1.489 | <.001 | 1.365 | 1.624 |
| Congenital Heart Disease | 0.955 | 0.656 | 0.779 | 1.171 |
| Connective Tissue Or Autoimmune Disease Excluding Lupus | 1.29 | <.001 | 1.245 | 1.337 |
| Current Tobacco Use | 1.384 | <.001 | 1.348 | 1.422 |
| Gastrointestinal Disease Crohns Disease | 1.127 | <.001 | 1.096 | 1.16 |
| Gonococcal | 1.809 | <.001 | 1.541 | 2.123 |
| Group B Strep | 0.536 | <.001 | 0.525 | 0.547 |
| Hepatitis | 1.12 | <.001 | 1.053 | 1.19 |
| Herpes Simplex | 0.874 | <.001 | 0.843 | 0.907 |
| History Tobacco Use | 1.03 | 0.024 | 1.004 | 1.056 |
| HIV/AIDS | 1.111 | 0.221 | 0.938 | 1.316 |
| Hyperthyroidism Graves Disease | 1.252 | <.001 | 1.149 | 1.364 |
| Mental Health | 1.266 | <.001 | 1.242 | 1.29 |
| Neuromuscular Disease | 1.122 | <.001 | 1.075 | 1.172 |
| Obesity | 0.837 | <.001 | 0.822 | 0.853 |
| Other STI | 1.066 | 0.161 | 0.975 | 1.166 |
| Polycystic Ovary Syndrome | 1.4 | <.001 | 1.323 | 1.482 |
| Pre-existing Hypertension | 1.128 | <.001 | 1.093 | 1.163 |
| Previous Cesarean | 1.01 | 0.237 | 0.993 | 1.028 |
| Prior Fetal Death | 1.486 | <.001 | 1.345 | 1.642 |
| Pulmonary Hypertension | 1.244 | 0.095 | 0.963 | 1.609 |
| Seizure Disorders | 1.181 | <.001 | 1.096 | 1.271 |
| Sickle Cell | 0.984 | 0.627 | 0.922 | 1.05 |
| Substance Use | 1.703 | <.001 | 1.653 | 1.754 |
| Supervision Of High Risk Pregnancy | 1.535 | <.001 | 1.422 | 1.657 |
| Syphilis | 1.623 | <.001 | 1.462 | 1.801 |
| Systemic Lupus Erythematosus | 1.211 | 0.003 | 1.066 | 1.377 |
| Trichomoniasis | 1.892 | <.001 | 1.722 | 2.077 |
| Type I Pre-existing Diabetes | 2.484 | <.001 | 2.284 | 2.7 |
| Type II Pre-existing Diabetes | 1.704 | <.001 | 1.605 | 1.809 |
| Uterine Fibroids | 1.223 | <.001 | 1.173 | 1.275 |

Supplemental Table 12. Cesarean Delivery Regression Coefficients

| **term** | **odds.ratio** | **p.value** | **conf.low** | **conf.high** |
| --- | --- | --- | --- | --- |
| (Intercept) | 0.251 | <.001 | 0.229 | 0.275 |
| COVID-19: Alpha | 1.076 | 0.016 | 1.014 | 1.143 |
| COVID-19: Delta | 1.202 | <.001 | 1.142 | 1.264 |
| COVID-19: Omicron #1 | 1.048 | 0.014 | 1.01 | 1.089 |
| COVID-19: Omicron #2 | 1.165 | <.001 | 1.099 | 1.234 |
| COVID-19: Pre-Alpha | 1.1 | <.001 | 1.05 | 1.152 |
| Age: 10-19 | 0.968 | <.001 | 0.951 | 0.985 |
| Age: 20-24 | 0.983 | 0.001 | 0.974 | 0.993 |
| Age: 25-29 | 0.972 | <.001 | 0.964 | 0.981 |
| Age: 35-39 | 1.113 | <.001 | 1.103 | 1.124 |
| Age: 40-44 | 1.423 | <.001 | 1.398 | 1.449 |
| Age: 45-64 | 2.701 | <.001 | 2.539 | 2.874 |
| Race: American Indian | 0.99 | 0.549 | 0.956 | 1.024 |
| Race: Asian | 1.085 | <.001 | 1.068 | 1.102 |
| Race: Black | 1.085 | <.001 | 1.074 | 1.096 |
| Race: Other | 0.941 | <.001 | 0.93 | 0.953 |
| Race: Pacific Islander | 1.017 | 0.351 | 0.982 | 1.052 |
| Race: Unable To Determine | 0.994 | 0.399 | 0.979 | 1.008 |
| Ethnicity: Hispanic Or Latino | 1.041 | <.001 | 1.031 | 1.05 |
| Ethnicity: Unknown | 1.065 | <.001 | 1.053 | 1.077 |
| Payor: Charity Indigent | 0.743 | <.001 | 0.668 | 0.827 |
| Payor: Medicaid | 0.751 | <.001 | 0.745 | 0.756 |
| Payor: Medicare | 0.904 | <.001 | 0.865 | 0.944 |
| Payor: Other Unknown | 0.8 | <.001 | 0.788 | 0.812 |
| Admission Type: Information Not Available | 0.867 | <.001 | 0.852 | 0.881 |
| Admission Type: Trauma Center/Emergency/Urgent | 0.751 | <.001 | 0.746 | 0.756 |
| Admission Source: Clinic | 1.169 | <.001 | 1.159 | 1.179 |
| Admission Source: Information Not Available/Other | 0.863 | <.001 | 0.837 | 0.889 |
| Admission Source: Transfer | 1.651 | <.001 | 1.607 | 1.695 |
| Acquired Heart Disease | 1.223 | <.001 | 1.206 | 1.241 |
| Alcohol Use | 1.091 | 0.118 | 0.978 | 1.218 |
| Anemia | 1.031 | <.001 | 1.021 | 1.042 |
| Asthma | 1.083 | <.001 | 1.07 | 1.097 |
| Bariatric Surgery | 1.05 | 0.015 | 1.01 | 1.093 |
| Pre-existing Bleeding Disorder | 1.249 | <.001 | 1.227 | 1.272 |
| Cancer | 1.156 | <.001 | 1.108 | 1.207 |
| Cardiac Valvular Disease | 1.068 | 0.107 | 0.986 | 1.158 |
| Chlamydia | 0.92 | 0.017 | 0.859 | 0.985 |
| Chronic Ischemic Heart Disease | 1.209 | 0.011 | 1.044 | 1.4 |
| Chronic Renal Disease | 2.026 | <.001 | 1.916 | 2.142 |
| Congenital Heart Disease | 1.187 | 0.001 | 1.073 | 1.312 |
| Connective Tissue Or Autoimmune Disease Excluding Lupus | 1.643 | <.001 | 1.612 | 1.673 |
| Current Tobacco Use | 1.085 | <.001 | 1.067 | 1.103 |
| Gastrointestinal Disease Crohns Disease | 1.346 | <.001 | 1.327 | 1.366 |
| Gonococcal | 0.838 | 0.019 | 0.724 | 0.971 |
| Group B Strep | 0.739 | <.001 | 0.733 | 0.745 |
| Hepatitis | 1.088 | <.001 | 1.046 | 1.131 |
| Herpes Simplex | 1.125 | <.001 | 1.106 | 1.145 |
| History Tobacco Use | 1.087 | <.001 | 1.073 | 1.101 |
| HIV/AIDS | 1.548 | <.001 | 1.398 | 1.714 |
| Hyperthyroidism Graves Disease | 0.943 | 0.017 | 0.899 | 0.99 |
| Mental Health | 1.126 | <.001 | 1.115 | 1.137 |
| Neuromuscular Disease | 1.166 | <.001 | 1.139 | 1.193 |
| Obesity | 1.815 | <.001 | 1.8 | 1.83 |
| Other STI | 1 | 0.992 | 0.953 | 1.049 |
| Polycystic Ovary Syndrome | 1.405 | <.001 | 1.365 | 1.445 |
| Pre-existing Hypertension | 1.467 | <.001 | 1.444 | 1.491 |
| Previous Cesarean | 21.715 | <.001 | 21.516 | 21.916 |
| Prior Fetal Death | 0.997 | 0.92 | 0.94 | 1.058 |
| Pulmonary Hypertension | 2.041 | <.001 | 1.687 | 2.47 |
| Seizure Disorders | 1.112 | <.001 | 1.065 | 1.161 |
| Sickle Cell | 1.041 | 0.03 | 1.004 | 1.079 |
| Substance Use | 1.034 | 0.001 | 1.014 | 1.056 |
| Supervision Of High Risk Pregnancy | 1.053 | 0.034 | 1.004 | 1.105 |
| Syphilis | 1.164 | <.001 | 1.076 | 1.26 |
| Systemic Lupus Erythematosus | 1.192 | <.001 | 1.108 | 1.281 |
| Trichomoniasis | 1.011 | 0.785 | 0.935 | 1.094 |
| Type I Pre-existing Diabetes | 4.132 | <.001 | 3.889 | 4.39 |
| Type II Pre-existing Diabetes | 2.38 | <.001 | 2.289 | 2.474 |
| Uterine Fibroids | 4.502 | <.001 | 4.409 | 4.598 |

Supplemental Table 13. Cost Regression Coefficients

| **term** | **odds.ratio** | **p.value** | **conf.low** | **conf.high** |
| --- | --- | --- | --- | --- |
| (Intercept) | 8565.814 | <.001 | 8285.952 | 8855.129 |
| COVID-19: Alpha | 1.088 | <.001 | 1.073 | 1.102 |
| COVID-19: Delta | 1.174 | <.001 | 1.161 | 1.187 |
| COVID-19: Omicron #1 | 1.052 | <.001 | 1.044 | 1.061 |
| COVID-19: Omicron #2 | 1.082 | <.001 | 1.068 | 1.096 |
| COVID-19: Pre-Alpha | 1.111 | <.001 | 1.1 | 1.122 |
| Age: 10-19 | 1.055 | <.001 | 1.051 | 1.059 |
| Age: 20-24 | 1.012 | <.001 | 1.01 | 1.014 |
| Age: 25-29 | 0.993 | <.001 | 0.992 | 0.995 |
| Age: 35-39 | 1.029 | <.001 | 1.026 | 1.031 |
| Age: 40-44 | 1.085 | <.001 | 1.081 | 1.09 |
| Age: 45-64 | 1.213 | <.001 | 1.196 | 1.231 |
| Race: American Indian | 0.985 | <.001 | 0.978 | 0.992 |
| Race: Asian | 1.073 | <.001 | 1.07 | 1.077 |
| Race: Black | 1.057 | <.001 | 1.055 | 1.06 |
| Race: Other | 1.045 | <.001 | 1.042 | 1.048 |
| Race: Pacific Islander | 1.01 | 0.007 | 1.003 | 1.017 |
| Race: Unable To Determine | 1.016 | <.001 | 1.013 | 1.019 |
| Ethnicity: Hispanic Or Latino | 1.018 | <.001 | 1.016 | 1.021 |
| Ethnicity: Unknown | 0.91 | <.001 | 0.907 | 0.912 |
| Payor: Charity Indigent | 0.99 | 0.358 | 0.968 | 1.012 |
| Payor: Medicaid | 0.956 | <.001 | 0.954 | 0.958 |
| Payor: Medicare | 0.999 | 0.905 | 0.99 | 1.009 |
| Payor: Other Unknown | 0.939 | <.001 | 0.936 | 0.942 |
| Admission Type: Information Not Available | 0.968 | <.001 | 0.965 | 0.972 |
| Admission Type: Trauma Center/Emergency/Urgent | 1.003 | <.001 | 1.002 | 1.005 |
| Admission Source: Clinic | 0.982 | <.001 | 0.98 | 0.983 |
| Admission Source: Information Not Available/Other | 1.02 | <.001 | 1.013 | 1.026 |
| Admission Source: Transfer | 1.261 | <.001 | 1.253 | 1.269 |
| Acquired Heart Disease | 1.139 | <.001 | 1.136 | 1.143 |
| Alcohol Use | 1.098 | <.001 | 1.071 | 1.125 |
| Anemia | 1.014 | <.001 | 1.012 | 1.017 |
| Asthma | 1.039 | <.001 | 1.036 | 1.041 |
| Bariatric Surgery | 1.009 | 0.055 | 1 | 1.018 |
| Pre-existing Bleeding Disorder | 1.143 | <.001 | 1.138 | 1.147 |
| Cancer | 1.043 | <.001 | 1.033 | 1.053 |
| Cardiac Valvular Disease | 1.045 | <.001 | 1.026 | 1.065 |
| Chlamydia | 1.016 | 0.025 | 1.002 | 1.031 |
| Chronic Ischemic Heart Disease | 1.051 | 0.003 | 1.017 | 1.087 |
| Chronic Renal Disease | 1.351 | <.001 | 1.334 | 1.369 |
| Congenital Heart Disease | 1.059 | <.001 | 1.034 | 1.083 |
| Connective Tissue Or Autoimmune Disease Excluding Lupus | 1.058 | <.001 | 1.054 | 1.063 |
| Current Tobacco Use | 1.009 | <.001 | 1.005 | 1.013 |
| Gastrointestinal Disease Crohns Disease | 1.103 | <.001 | 1.099 | 1.107 |
| Gonococcal | 1.045 | 0.005 | 1.014 | 1.076 |
| Group B Strep | 0.997 | 0.005 | 0.996 | 0.999 |
| Hepatitis | 1.055 | <.001 | 1.046 | 1.064 |
| Herpes Simplex | 1.011 | <.001 | 1.007 | 1.015 |
| History Tobacco Use | 1.015 | <.001 | 1.012 | 1.018 |
| HIV/AIDS | 1.129 | <.001 | 1.104 | 1.155 |
| Hyperthyroidism Graves Disease | 1.023 | <.001 | 1.012 | 1.034 |
| Mental Health | 1.059 | <.001 | 1.057 | 1.061 |
| Neuromuscular Disease | 1.103 | <.001 | 1.098 | 1.109 |
| Obesity | 1.11 | <.001 | 1.108 | 1.113 |
| Other STI | 1.015 | 0.007 | 1.004 | 1.025 |
| Polycystic Ovary Syndrome | 1.073 | <.001 | 1.066 | 1.08 |
| Pre-existing Hypertension | 1.153 | <.001 | 1.148 | 1.157 |
| Previous Cesarean | 1.098 | <.001 | 1.096 | 1.1 |
| Prior Fetal Death | 1.048 | <.001 | 1.034 | 1.062 |
| Pulmonary Hypertension | 1.474 | <.001 | 1.411 | 1.539 |
| Seizure Disorders | 1.006 | 0.245 | 0.996 | 1.016 |
| Sickle Cell | 1.016 | <.001 | 1.008 | 1.024 |
| Substance Use | 1.042 | <.001 | 1.037 | 1.046 |
| Supervision Of High Risk Pregnancy | 1.062 | <.001 | 1.051 | 1.073 |
| Syphilis | 1.111 | <.001 | 1.092 | 1.131 |
| Systemic Lupus Erythematosus | 1.069 | <.001 | 1.052 | 1.087 |
| Trichomoniasis | 1.097 | <.001 | 1.078 | 1.116 |
| Type I Pre-existing Diabetes | 1.389 | <.001 | 1.37 | 1.409 |
| Type II Pre-existing Diabetes | 1.214 | <.001 | 1.204 | 1.225 |
| Uterine Fibroids | 1.214 | <.001 | 1.208 | 1.22 |

Supplemental Table 14. Length of Stay Regression Coefficients

| **term** | **exp.estimate** | **p.value** | **conf.low** | **conf.high** |
| --- | --- | --- | --- | --- |
| (Intercept) | 2.155 | <.001 | 2.071 | 2.243 |
| COVID-19: Alpha | 1.061 | <.001 | 1.046 | 1.076 |
| COVID-19: Delta | 1.113 | <.001 | 1.099 | 1.126 |
| COVID-19: Omicron #1 | 1.02 | <.001 | 1.011 | 1.03 |
| COVID-19: Omicron #2 | 1.051 | <.001 | 1.036 | 1.066 |
| COVID-19: Pre-Alpha | 1.037 | <.001 | 1.025 | 1.049 |
| Age: 10-19 | 1.102 | <.001 | 1.098 | 1.107 |
| Age: 20-24 | 1.036 | <.001 | 1.033 | 1.038 |
| Age: 25-29 | 1.007 | <.001 | 1.005 | 1.009 |
| Age: 35-39 | 1.02 | <.001 | 1.018 | 1.023 |
| Age: 40-44 | 1.08 | <.001 | 1.076 | 1.085 |
| Age: 45-64 | 1.202 | <.001 | 1.185 | 1.22 |
| Race: American Indian | 1.044 | <.001 | 1.035 | 1.052 |
| Race: Asian | 1.062 | <.001 | 1.058 | 1.066 |
| Race: Black | 1.064 | <.001 | 1.062 | 1.067 |
| Race: Other | 1.023 | <.001 | 1.02 | 1.027 |
| Race: Pacific Islander | 1.059 | <.001 | 1.05 | 1.068 |
| Race: Unable To Determine | 1.03 | <.001 | 1.026 | 1.034 |
| Ethnicity: Hispanic Or Latino | 0.997 | 0.015 | 0.995 | 0.999 |
| Ethnicity: Unknown | 1 | 0.759 | 0.998 | 1.003 |
| Payor: Charity Indigent | 0.988 | 0.37 | 0.963 | 1.014 |
| Payor: Medicaid | 0.958 | <.001 | 0.956 | 0.959 |
| Payor: Medicare | 1.004 | 0.418 | 0.994 | 1.015 |
| Payor: Other Unknown | 0.95 | <.001 | 0.946 | 0.953 |
| Admission Type: Information Not Available | 1.006 | 0.008 | 1.001 | 1.01 |
| Admission Type: Trauma Center/Emergency/Urgent | 1.019 | <.001 | 1.017 | 1.021 |
| Admission Source: Clinic | 1.022 | <.001 | 1.019 | 1.024 |
| Admission Source: Information Not Available/Other | 0.994 | 0.089 | 0.986 | 1.001 |
| Admission Source: Transfer | 1.329 | <.001 | 1.321 | 1.337 |
| Acquired Heart Disease | 1.11 | <.001 | 1.106 | 1.113 |
| Alcohol Use | 1.091 | <.001 | 1.063 | 1.119 |
| Anemia | 1.025 | <.001 | 1.023 | 1.028 |
| Asthma | 1.034 | <.001 | 1.031 | 1.037 |
| Bariatric Surgery | 1.001 | 0.818 | 0.992 | 1.011 |
| Pre-existing Bleeding Disorder | 1.121 | <.001 | 1.116 | 1.125 |
| Cancer | 1.042 | <.001 | 1.031 | 1.053 |
| Cardiac Valvular Disease | 1.053 | <.001 | 1.034 | 1.073 |
| Chlamydia | 1.027 | 0.001 | 1.011 | 1.043 |
| Chronic Ischemic Heart Disease | 1.044 | 0.009 | 1.011 | 1.079 |
| Chronic Renal Disease | 1.318 | <.001 | 1.302 | 1.334 |
| Congenital Heart Disease | 1.057 | <.001 | 1.031 | 1.083 |
| Connective Tissue Or Autoimmune Disease Excluding Lupus | 1.051 | <.001 | 1.046 | 1.056 |
| Current Tobacco Use | 0.991 | <.001 | 0.987 | 0.995 |
| Gastrointestinal Disease Crohns Disease | 1.093 | <.001 | 1.089 | 1.096 |
| Gonococcal | 1.068 | <.001 | 1.034 | 1.103 |
| Group B Strep | 1.008 | <.001 | 1.006 | 1.011 |
| Hepatitis | 1.072 | <.001 | 1.062 | 1.082 |
| Herpes Simplex | 1.01 | <.001 | 1.005 | 1.014 |
| History Tobacco Use | 1.012 | <.001 | 1.009 | 1.015 |
| HIV/AIDS | 1.106 | <.001 | 1.08 | 1.132 |
| Hyperthyroidism Graves Disease | 1.02 | 0.001 | 1.008 | 1.032 |
| Mental Health | 1.073 | <.001 | 1.07 | 1.075 |
| Neuromuscular Disease | 1.104 | <.001 | 1.098 | 1.11 |
| Obesity | 1.122 | <.001 | 1.119 | 1.124 |
| Other STI | 1.007 | 0.285 | 0.995 | 1.019 |
| Polycystic Ovary Syndrome | 1.074 | <.001 | 1.067 | 1.082 |
| Pre-existing Hypertension | 1.179 | <.001 | 1.174 | 1.183 |
| Previous Cesarean | 1.002 | 0.031 | 1 | 1.004 |
| Prior Fetal Death | 1.048 | <.001 | 1.033 | 1.063 |
| Pulmonary Hypertension | 1.379 | <.001 | 1.33 | 1.429 |
| Seizure Disorders | 1 | 0.956 | 0.989 | 1.01 |
| Sickle Cell | 1.028 | <.001 | 1.019 | 1.037 |
| Substance Use | 1.048 | <.001 | 1.043 | 1.053 |
| Supervision Of High Risk Pregnancy | 1.021 | 0.001 | 1.009 | 1.033 |
| Syphilis | 1.121 | <.001 | 1.101 | 1.142 |
| Systemic Lupus Erythematosus | 1.089 | <.001 | 1.071 | 1.108 |
| Trichomoniasis | 1.106 | <.001 | 1.087 | 1.126 |
| Type I Pre-existing Diabetes | 1.432 | <.001 | 1.414 | 1.451 |
| Type II Pre-existing Diabetes | 1.235 | <.001 | 1.225 | 1.246 |
| Uterine Fibroids | 1.18 | <.001 | 1.174 | 1.186 |
