# Supplementary material for: Association between COVID-19 by variant period and delivery-related outcomes in the United States
Source: PLoS One. 2026 Aug 12;21(8):e0355223. doi: 10.1371/journal.pone.0355223 (PMC13466025; doi:10.1371/journal.pone.0355223)
Supplement: Supplementary file 6 [file pone.0355223.s009.docx]

**Supplementary Appendix:** **Regression Models for Patient Outcomes with COVID-19 Variant Period Indicators**

This study evaluates the impact of different COVID-19 infection occurring within each variant period on patient outcomes, stratified by multiple health outcomes. These outcomes include binary outcomes (e.g., presence of complications), continuous outcomes (e.g., length of stay), and 90-day healthcare costs. The data used in this analysis were derived from a cohort of patients, with each patient assigned to one of several COVID-19 variant periods during the study period.

For binary outcomes, logistic regression models were used to estimate the probability of event $Y_{ij}^{(k)}$ occurring for patient i in hospital j during for outcome k. Each outcome variable was dichotomous, indicating the presence or absence of a specific event (e.g., complication, mortality). The logistic regression model was specified as follows:

$$\log(P(Y_{ij}^{(k)}=1))= \beta_{0}^{(k)}+ \sum_{v=1}^{5} \beta_{v}^{(k)}\cdot{COVID}_{v, ij}+ X_{ij}\gamma^{\left( k \right)} + Z_{j}\delta^{\left( k \right)} for each outcome k$$

Where:

- $P(Y_{ij}^{(k)}=1)$ represents the probability of the outcome k occurring for patient i in hospital j.
- $\beta_{0}^{(k)}$ is the intercept for outcome k.
- $\beta_{v}^{(k)}$ are the coefficients for each COVID-19 variant v, where v ranges from 1 to 5, representing the different COVID-19 variants.
- ${COVID}_{v, ij}$ is an indicator variable for whether patient i in hospital j is exposed to COVID-19 variant v.
- $X_{ij}$ represents patient-level covariates, including demographic and clinical factors (e.g., age, comorbidities).
- $\gamma^{\left( k \right)}$ are the coefficients associated with patient-level covariates for outcome k.
- $Z_{j}$ represents hospital-level factors (e.g., hospital type, region).
- $\delta^{\left( k \right)}$ are the coefficients for hospital-level covariates for outcome k.

This model was estimated separately for each outcome k, where k corresponds to the different patient outcomes (e.g., complications, mortality, etc.).

For continuous outcomes such as delivery-related metrics and length of stay (LOS), a Gamma regression model with a log link function was employed to account for the right-skewed distribution of these outcomes. The model was specified as follows:

$$\log(\mathbb{E}\left( Y_{ij}^{\left( k \right)} \right))= \beta_{0}^{(k)}+ \sum_{v=1}^{5} \beta_{v}^{(k)}\cdot{COVID}_{v, ij}+ X_{ij}\gamma^{\left( k \right)}+ Z_{j}\delta^{\left( k \right)} for each outcome k$$

Where:

- $\mathbb{E}\left( Y_{ij}^{\left( k \right)} \right)$ is the expected value of the outcome $Y_{ij}^{\left( k \right)}$, representing the delivery-related measure or LOS for patient i in hospital j for outcome k.
- The other terms are defined as in the binary outcome model.

For the 90-day healthcare cost model, a similar Gamma regression model with a log link was used, augmented by the inclusion of the Consumer Price Index (CPI) to account for inflationary effects over time. The model was specified as:

$$\log(\mathbb{E}\left( Y_{ij}^{\left( k \right)} \right))= \beta_{0}^{(k)}+ \sum_{v=1}^{5} \beta_{v}^{(k)}{COVID}_{v, ij}+ X_{ij}\gamma^{\left( k \right)}+ Z_{j}\delta^{\left( k \right)}+ {CPI}_{t}\phi^{(k)} for each outcome k$$

Where:

- ${CPI}_{t}$represents the Consumer Price Index for the relevant time period t.
- $\phi^{(k)}$ is the coefficient associated with CPI for outcome k.
- The other terms are defined as in the previous models.
